# I-GraphECG: Observability-Based Lead Selection and Interpretable Disease Prediction from the 12-Lead ECG Using a Gray-Box Graph Electrophysiology Surrogate

**DOI:** 10.3390/s26185933

**Published:** 2026-09-19

**Authors:** Limin Zhao, Hongtao Xu, Pengjian Wang, Weicheng Fu, Ningning Zhang

**Affiliations:** School of Electronic Information and Electrical Engineering, Tianshui Normal University, Tianshui 741001, China; sqxuhontao@163.com (H.X.); wangpengjian628@126.com (P.W.); fuweicheng@tsnu.edu.cn (W.F.); 18909461746@163.com (N.Z.)

**Keywords:** electrocardiogram, wearable ECG, interpretable machine learning, graph electrophysiology, observability, optimal sensor selection

## Abstract

Wearable ECG increasingly records fewer than the standard 12 leads, so a model must be accurate, interpretable, and explicit about the information lost under lead reduction. I-GraphECG is a gray-box graph electrophysiology surrogate: an encoder maps a 12-lead median beat to a bounded vector of 47 equivalent electrophysiological descriptors, a constrained eight-node conduction-graph decoder reconstructs the signal, and disease prediction uses the descriptors rather than the waveform. On a clean PTB-XL four-class subset, median beats are reconstructed at median correlation 0.91, and macro-AUROC reaches ≈0.90, near the black-box references (0.920–0.924). Myocardial infarction is under-detected (recall ≈ 0.49), consistent with the ST-source’s low observability, so the model must not be used as a stand-alone rule-out for infarction. Observability-based sensor selection makes explicit what each lead set can resolve: lead removal provably cannot lower any Cramér–Rao bound; under the data-derived noise models, every optimal three-lead set retains one of the precordial leads V2–V5, and the conventional reduced set is never observability-optimal across seven noise models, though the exact montage is noise-model-dependent. Reconstruction and the observability analysis of the fixed model transfer zero-shot to US and Chinese cohorts; parameter-only disease prediction degrades externally, and infarction, untestable in those cohorts, remains internally demonstrated.

## 1. Introduction

The electrocardiogram (ECG) remains one of the most widely deployed biomedical sensing modalities. The standard 12-lead ECG provides multiple spatial projections of cardiac electrical activity and is used routinely to detect myocardial infarction, conduction abnormalities, and repolarization changes. In parallel, ambulatory and wearable ECG sensors have made long-duration monitoring increasingly accessible, but such devices typically record fewer leads and therefore sample less of the spatial information carried by a full clinical ECG. A model for such sensing should offer predictive accuracy, physiological interpretability, and an explicit account of the information lost under lead reduction; existing models rarely provide all three.

Deep-learning models have achieved strong performance in ECG classification, including cardiologist-level arrhythmia detection and automatic diagnosis from 12-lead ECGs [1,2,3]. However, their internal representations are typically not aligned with clinically meaningful electrophysiological concepts such as conduction timing, repolarization dynamics, or lead-dependent projection effects. Because such models are trained as direct mappings from waveform to label, the same diagnostic outcome may be reached through representations that do not correspond to identifiable physiological quantities.

Mechanistic cardiac electrophysiology models—bidomain, monodomain, reaction–diffusion, and digital-twin formulations [4,5,6,7]—provide detailed biophysical interpretation, but inverse estimation from a routine 12-lead ECG is ill-posed: standard leads sample cardiac electrical activity only sparsely, so many internal activation or repolarization patterns produce similar body-surface signals, and unique recovery of detailed ionic, anatomical, or tissue-conductivity parameters is not generally attainable without additional imaging, body-surface mapping, or strong priors. The two paradigms thus trade predictive performance against physiological interpretability.

**Figure 1:**
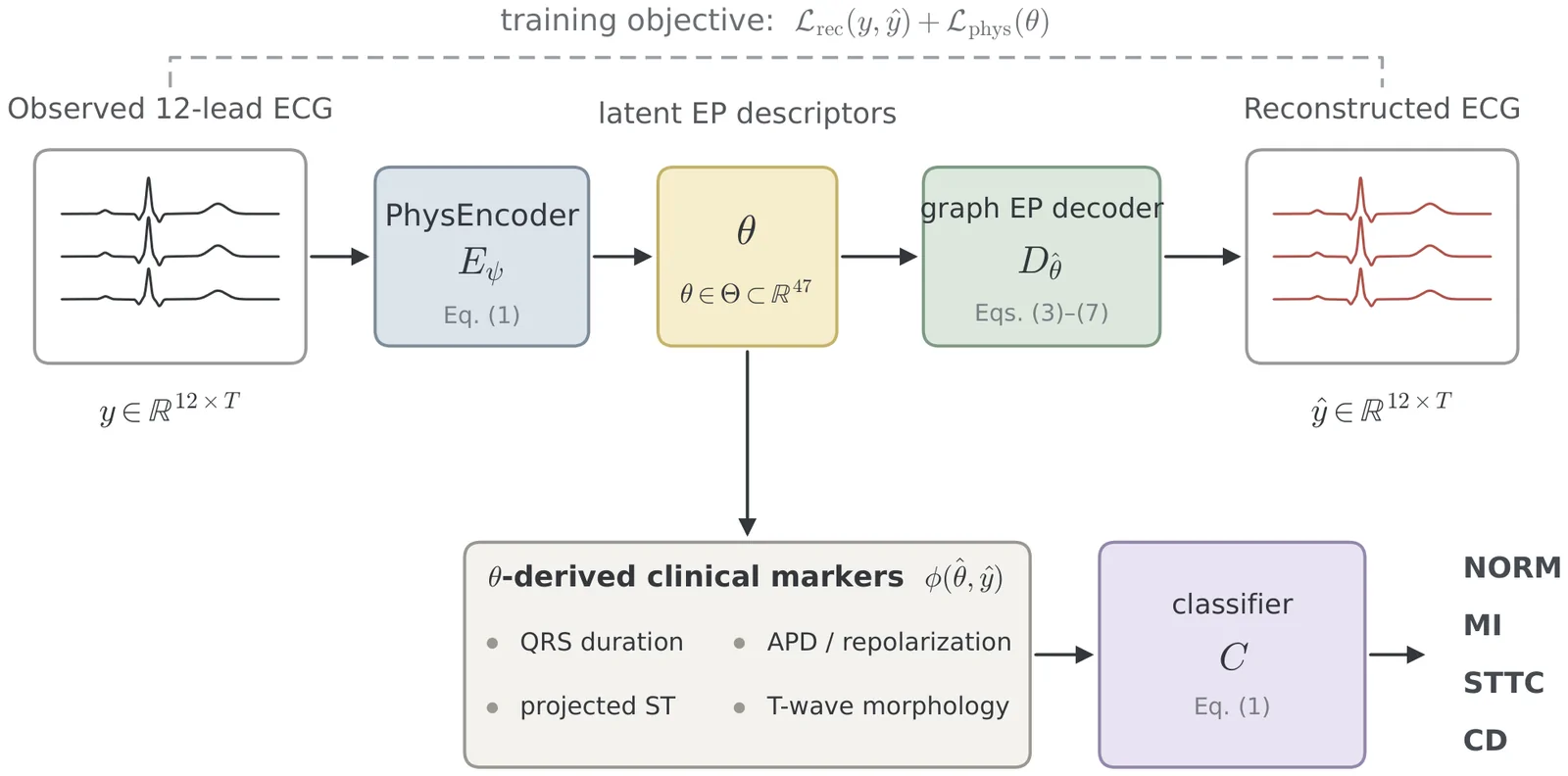
Overview of I-GraphECG. An observed 12-lead median-beat ECG y(t) is mapped by the encoder $E_{\psi }$ (an amortized approximation to a constrained parameter estimator) to a bounded set of equivalent electrophysiological descriptors $\hat{\theta }\in R^{47}$ (Equation (1)). The descriptors drive a graph electrophysiology decoder $D_{\hat{\theta }}$ (Equations (3)–(7)) in which activation times propagate along an explicit directed conduction graph ($\delta_{j}=\delta_{\mathrm{root}}+\sum_{e\in \mathrm{path}(j)}d_{e}$), generate per-node double-sigmoid waveforms, and are projected to the body surface through a constrained low-rank lead-field (H=AB, rank three) to reconstruct the 12-lead signal $\hat{y}\left(t\right)$ under the reconstruction constraint $\hat{y}\approx y$. Disease prediction uses a classifier *C* (Equation (1)) acting on interpretable descriptors $\phi (\hat{\theta },\hat{y})$—conduction (QRS-duration), repolarization (APD), T-wave morphology, and lead-projected ST—rather than on the raw waveform.

We formulate ECG diagnosis as a structured sensor-to-state inverse problem: instead of mapping waveforms directly to labels, I-GraphECG maps a 12-lead median-beat ECG to a bounded set of equivalent electrophysiological descriptors. The descriptors drive an eight-node graph electrophysiology surrogate in which eikonal-inspired activation times propagate along an explicit directed conduction graph and a constrained low-rank lead-field projects the internal sources to the body surface; the decoder predicts the independent leads I, II, and V1–V6 directly, and III, aVR, aVL, and aVF are obtained from the Einthoven–Goldberger relations. Disease prediction is then performed from descriptors derived from this structured representation. The central hypothesis is that a 12-lead ECG can be represented by a compact set of equivalent electrophysiological descriptors that are simultaneously reconstructive and discriminative, enabling diagnostic prediction, physiological interpretation, and lead-set analysis within one framework. Figure 1 summarizes the proposed framework.

### 1.1. Contributions

1. We propose I-GraphECG, a low-order gray-box *graph electrophysiology* surrogate that maps a 12-lead ECG median beat to bounded equivalent electrophysiological descriptors and reconstructs the observed signal under physiological constraints.
2. We formulate the encoder (PhysEncoder) as an amortized approximation to a constrained batch parameter estimator, trained self-supervised on recorded ECGs rather than on simulator output, yielding a structured, bounded parameterization whose stably recoverable (observable) subspace is low-dimensional, rather than an unconstrained latent embedding; the amortization gap is measured (Section 3.2).
3. We introduce interpretable disease descriptors—conduction (QRS-duration), repolarization and T-wave morphology, and lead-projected ST—and assess their stability across training seeds, showing which of them recover known electrophysiological signatures across NORM, MI, STTC, and CD.
4. We cast reduced-lead design as an observability-based optimal sensor-selection problem on the Fisher information of the surrogate’s physiological parameters, population-averaged over recorded ECGs and restricted to standard 12-lead subsets; the selection inherits the submodular (1−1/e) guarantee of greedy log det selection [8], and a lead-field singular-value (low-gain-direction) account explains which parameters each lead set can resolve—directly relevant to wearable and reduced-lead ECG sensing.

### 1.2. Scope

I-GraphECG operates on a single median beat sampled at 100 Hz. It is, therefore, a model of *beat morphology*: it represents the repeatable QRS–T shape and the spatial way that shape projects onto leads; the P-wave is not a modeling target (Section 3.2), and it does not represent rhythm, beat-to-beat variability, or arrhythmia, while the 100 Hz rate attenuates high-frequency QRS detail such as fragmentation and notching. The intended use follows from this: morphological ECG interpretation and sensor- or lead-set design, with disease probabilities as decision support requiring clinical confirmation. It is not a general-purpose ECG diagnostic system, and the classes it distinguishes here—including conduction disturbance, which PTB-XL diagnoses predominantly from beat morphology and interval measurements that a median beat preserves—were chosen to be resolvable within that scope (Section 4.5).

### 1.3. Related Work

#### 1.3.1. Black-Box 12-Lead ECG Classification

Deep networks map raw 12-lead ECG to diagnostic labels with high accuracy [1,2,3], increasingly through large self-supervised foundation models [9]. These models set the performance reference we compare against (Section 2.1); I-GraphECG is instead a parameter-inversion model whose intermediate state is physiologically organized.

#### 1.3.2. Interpretable ECG Analysis

Post-hoc saliency and attention methods indicate which samples or leads influence a prediction, but they explain a black box rather than provide a mechanistic state. In I-GraphECG, interpretability instead arises from estimated equivalent electrophysiological descriptors and a constrained reconstruction. Recent explainable-ECG work adds post-hoc or attention-based transparency to high-accuracy classifiers, including on PTB-XL [10]. Unlike concept-bottleneck models [11], whose concepts are supervised discrete labels, and unlike the variational auto-encoder factors of FactorECG [12], which also reconstruct the 12-lead median beat but carry no fixed physical meaning, our bottleneck is a set of continuous electrophysiological parameters with units and bounds that must reconstruct the signal through a fixed, physically constrained decoder. The closest low-order parametric precedent is the five-dipole 3D-FMM model of Rueda et al. [13], whose per-wave oscillator parameters serve as diagnostic markers; I-GraphECG instead generates the waveform from conduction-graph delays, action-potential durations, and time constants under an eikonal-type propagation rule, amortizes the inverse with an encoder trained on PTB-XL, and analyses the parameters for identifiability and lead selection. Physics-informed and EP-PINN approaches [14,15] instead solve spatiotemporal field inversion or forward problems, whereas I-GraphECG fits a low-order, identifiability-aware surrogate aimed at observability analysis.

#### 1.3.3. Reduced-Lead Reconstruction and Wearable Sensing

A growing body of work reconstructs the full 12-lead ECG from fewer leads for wearable deployment [16,17,18,19], and lead subsets have also been chosen for classification performance directly [20]. Information-criterion selection of measurement sites has a long history in body-surface potential mapping, where limited lead sets were chosen to estimate the full map [21], including for wearable lead systems [22], and electrodes have been selected greedily by determinant and conditioning criteria of a dipole transfer matrix [23]. Those criteria act on a fixed linear forward model whose target is the potential map or a dipole; here the information is the Fisher information of a learned nonlinear surrogate’s 47 physiological parameters, averaged over recorded ECGs and restricted to subsets of the standard leads.

#### 1.3.4. ECG Inverse Problems and Cardiac Digital Twins

Bidomain, monodomain, and reaction–eikonal models provide detailed forward electrophysiology [4,6,24]. Eikonal-based cardiac digital twins personalize ventricular activation to the clinical 12-lead ECG together with patient imaging [7,25,26], and this line has itself exposed the non-identifiability of conduction-system properties from surface ECG [27]; neural regressors that map the ECG to simulator parameters have been trained on simulated data [28,29]. I-GraphECG does not attempt anatomical inversion: it replaces patient anatomy with a fixed eight-node graph with a learned lead-field, trains the inverse self-supervised on recorded ECGs, and turns the identifiability question into a lead-selection criterion.

## 2. Materials and Methods

### 2.1. Dataset, Diagnostic Labels, and Preprocessing

We used PTB-XL, a large publicly available clinical 12-lead ECG dataset [30], distributed through PhysioNet [31]. We focused on a clean four-class subset with diagnostic superclasses NORM, MI, STTC, and CD. Records were retained only when they matched exactly one of the four superclasses; records with overlapping hypertrophy labels were excluded from the main analysis to avoid confounding voltage, axis, and lead-field effects with conduction, repolarization, and ST-source effects. The final subset contained 15,709 of 21,799 records (Supplementary Note S1 gives the full disposition). Retention is strongly class-dependent—95.3% of records carrying NORM survive the filter against 46.3% for MI—so the criterion raises the NORM:MI ratio from 1.74 in the full database to 3.58 in the analyzed subset. Overlapping diagnoses would confound the identifiability analysis that is the study’s purpose, but the criterion changes the class prior the classifier is fitted to, with consequences quantified in Section 3.5 and Section 4.5. Because this clean single-label subset is a different and easier task than the multi-label PTB-XL benchmark, the absolute AUROC values reported here are *not directly comparable* to published leaderboard numbers (Section 3.9). We followed the official PTB-XL fold split (folds 1–8 for training, fold 9 for validation, fold 10 for testing; Table 1).

ECGs were processed using the 100 Hz version of PTB-XL. We used 100 Hz single median beats: a median beat suppresses noise and isolates the repeatable morphology that the surrogate represents, and 100 Hz is the lower-rate release that aligns with prior PTB-XL benchmarks. Each record was filtered with a zero-phase 0.5–40 Hz band-pass filter. R-peaks were detected primarily on lead II using NeuroKit2 [32], with fallback detection on V5 and multi-lead signals when required. For each record, we extracted R-aligned beats over −300 to +690 ms (100 samples) and computed a 12-lead median beat; this window includes the P-wave, which is not a modeling target (Section 3.2). Robust lead-wise scaling (median/IQR) was fit on the training set only and applied to validation and test records.

#### Black-Box Baselines

As an upper reference, we trained two black-box baselines on the same split: (i) a hand-crafted-feature gradient-boosted model (XGBoost [33]) using 111 morphological features extracted from the 12-lead median beat. Per lead, we computed the peak-to-peak amplitude, the R, Q, and S amplitudes and the R/S ratio, the ST-segment mean and slope over a 60–120 ms post-R window, and the T-wave amplitude and area over a 150–400 ms window; three global features were added: a vector-magnitude QRS-duration, the QT interval, and a Bazett QTc proxy at a fixed reference RR, since the median beat carries no rhythm information. Features were *z*-scored with statistics fit on the training fold only. (ii) A 1D ResNet [34] on the raw median-beat waveform. Together they define the black-box reference against which the interpretable model is compared.

### 2.2. Problem Formulation

#### Terminology

A parameter is *identifiable* if the Fisher information constrains it: its Cramér–Rao bound is small relative to its admissible range. A lead set is *observable* for a parameter if that parameter is identifiable from that lead set; observability is thus identifiability made relative to a sensor configuration. A descriptor is *interpretable* if it has a stated physiological reading, which is a claim about meaning and not about estimability—an interpretable descriptor can be poorly identifiable. Finally, we call the parameters *equivalent electrophysiological* rather than *physiological* quantities: they are lumped descriptors of an eight-node surrogate, not measurements of tissue properties.

Let $y\left(t\right)\in R^{12}$ denote the measured 12-lead median-beat ECG and $\theta \in R^{47}$ the equivalent electrophysiological descriptor vector. The model decomposes ECG analysis into parameter inversion, constrained reconstruction, and descriptor-based prediction:

(1) $$\hat{\theta }=E_{\psi }\left(y\right),\;\hat{y}\left(t\right)=D_{\hat{\theta }}\left(t\right),\;\hat{p}=C\;\left(\phi (\hat{\theta },\hat{y})\right),$$

where $E_{\psi }$ is the PhysEncoder, $D_{\hat{\theta }}$ is the graph electrophysiology decoder, ϕ(·) extracts interpretable features, and *C* is a conventional classifier trained on those features. The classifier does not operate on the raw waveform; it operates on the surrogate parameters and derived descriptors.

### 2.3. Eight-Node Graph Electrophysiology Surrogate (Eikonal-Inspired)

The heart was represented as an eight-node directed conduction graph G=(V,E) with coarse anatomical interpretation: node 0, right atrium/sinoatrial equivalent; node 1, atrioventricular/His; node 2, interventricular septum; and nodes 3–7, the right ventricular free wall, left ventricular anterior, lateral, and inferior walls, and apex. The directed edges form an anatomically motivated activation tree rooted at the sinoatrial node,

(2) E={(0→1),(1→2),(2→3),(2→4),(2→5),(2→6),(2→7)},

i.e., sinoatrial → atrioventricular/His → septum → ventricular walls/apex. This is a low-order, sensor-facing surrogate, not a patient-specific anatomical mesh.

#### 2.3.1. Activation by Graph Propagation (Eikonal Step)

The eight activation times are generated by propagation along *G* instead of being free parameters. With a root onset $\delta_{\mathrm{root}}$ and non-negative edge delays $d={\left(d_{e}\right)}_{e\in E}\in R_{\geq 0}^{7}$, the nodal activation times are

(3) $$\delta =\delta_{\mathrm{root}}\;1_{8}+M\;d,$$

where $M\in {\left\{0,1\right\}}^{8\times 7}$ is the fixed path-indicator matrix of the tree ($M_{je}=1$ if edge *e* lies on the root-to-*j* path). Because d≥0, the activation order $\delta_{\mathrm{child}}\geq \delta_{\mathrm{parent}}$ holds *by construction*; no soft activation-ordering penalty is required. Each $d_{e}$ is an interpretable conduction delay along a conduction-system segment, so a proximal conduction block raises the proximal delays and a fascicular block raises a specific leaf delay.

#### 2.3.2. Nodal Waveforms (Reaction Step)

For each node *i*, a double-sigmoid eikonal–APD waveform was used,

(4) $$U_{i}\left(t\right)=\sigma \;\left(\frac{t-\delta_{i}}{\tau_{\mathrm{dep},i}}\right)-\sigma \;\left(\frac{t-\delta_{i}-{\mathrm{APD}}_{i}}{\tau_{\mathrm{rep},i}}\right),$$

a unit-amplitude template whose amplitude is set by the nodal source gain $q_{i}$ and the global gain *g* below, with depolarization and repolarization time constants $\tau_{\mathrm{dep},i}$, $\tau_{\mathrm{rep},i}$ and equivalent action-potential duration ${\mathrm{APD}}_{i}$. The nodal source separated a fast depolarization/repolarization term from a slower ST-platform contribution,

(5) $$\begin{array}{cc} s_{i}\left(t\right) & =q_{i}\;\frac{dU_{i}\left(t\right)}{dt}+\alpha_{\mathrm{ST},i}\;P_{i}\left(t\right),\\ P_{i}\left(t\right) & =\sigma \;\left(\frac{t-\delta_{i}-0.04}{0.02}\right)\sigma \;\left(\frac{\delta_{i}+{\mathrm{APD}}_{i}-t}{0.04}\right), \end{array}$$

where $q_{i}$ is a nodal source gain and $\alpha_{\mathrm{ST},i}$ an ST-source term on ventricular nodes. The ST-platform window in $P_{i}\left(t\right)$ uses fixed offsets (a 40 ms onset delay and 20/40 ms edge widths) chosen to span the J-point-to-T region at 100 Hz; these are fixed hyperparameters, not fitted. The construction follows simplified excitation and reaction–eikonal formulations [24,35,36] in reduced form: the activation step is shortest-path propagation on a fixed tree, not an eikonal-PDE solve, and the reaction step is a double-sigmoid template, not a reaction–diffusion term. The nodal sources are generated independently and combined only through the lead-field below; the graph constrains the relative *timing* of activation, and coupled reaction–diffusion dynamics appear only in the auxiliary analysis of Section 2.10. The 47-dimensional bounded parameter vector was

(6) $$\begin{array}{cc} \theta & =[\delta_{\mathrm{root}},\;d_{1:7},\;{\mathrm{APD}}_{1:8},\;\tau_{\mathrm{dep},1:8},\;\tau_{\mathrm{rep},1:8},\;q_{1:8},\;\alpha_{\mathrm{ST},1:6},\;g],\\ \theta & =\theta_{\mathrm{center}}+\theta_{\mathrm{radius}}⊙\tanh\left(z\right), \end{array}$$

where *g* is a global gain, and the tanh map keeps every parameter within physiologically motivated bounds and enables boundary-rate analysis. The 47 parameters are chosen so that each has a physiological reading (per-segment conduction delays, per-node APD and time constants, ST-source, gains), not as a minimal sufficient statistic; the weakly identifiable directions are retained but regularized toward their centers, and only the high-observability directions are interpreted (Section 3.7).

### 2.4. Constrained Low-Rank Lead-Field Approximation

The body-surface ECG was generated from internal nodal sources through a low-rank lead-field. To limit the freedom of the projection, the decoder generated only the eight independent leads,

(7) $${\hat{y}}_{\mathrm{ind}}\left(t\right)=g\;H_{\mathrm{ind}}\;s\left(t\right),\;H_{\mathrm{ind}}=AB,\;A\in R^{8\times 3},\;B\in R^{3\times 8},$$

for I, II, and V1–V6; the rank-three factorization reflects the approximately dipolar mapping from cardiac sources to body-surface potentials [37]. The remaining limb leads were obtained from the standard Einthoven–Goldberger relations [37,38],

(8) $$\begin{array}{cccc} \mathrm{III} & =\mathrm{II}-I, & \mathrm{aVR} & =-\frac{I+\mathrm{II}}{2},\\ \mathrm{aVL} & =\frac{2\;I-\mathrm{II}}{2}, & \mathrm{aVF} & =\frac{2\;\mathrm{II}-I}{2}. \end{array}$$

The decoder operates in lead-wise robust-scaled coordinates $x_{\ell }=\left(x_{\ell }^{\mathrm{mV}}-m_{\ell }\right)/r_{\ell }$, where $m_{\ell }$ and $r_{\ell }$ are the training-set median and interquartile range of lead ℓ, so these relations are imposed not in millivolts but through their exact image in that space. Since the scaling is affine and the relations are linear, the image is affine: for a derived lead ℓ with physical coefficients $\left(a_{\ell },b_{\ell }\right)$ on (I, II),

(9) $$x_{\ell }=\underset{{\tilde{a}}_{\ell }}{\underset{︸}{\frac{a_{\ell }r_{I}}{r_{\ell }}}}x_{I}+\underset{{\tilde{b}}_{\ell }}{\underset{︸}{\frac{b_{\ell }r_{\mathrm{II}}}{r_{\ell }}}}x_{\mathrm{II}}+\underset{{\tilde{c}}_{\ell }}{\underset{︸}{\frac{a_{\ell }m_{I}+b_{\ell }m_{\mathrm{II}}-m_{\ell }}{r_{\ell }}}},$$

which reduces to the physical relation exactly when $r_{\ell }\equiv 1$ and $m_{\ell }\equiv 0$. For the locked training scalers, the coefficients $\left({\tilde{a}}_{\ell },{\tilde{b}}_{\ell },{\tilde{c}}_{\ell }\right)$ are listed in Table S10; they differ substantially from the physical values because the limb leads have unequal robust scales. After inverse scaling, the physical relations hold in millivolts to machine precision, whereas applying the physical coefficients to scaled signals inflates the derived-lead errors roughly threefold (Table S10); since the waveform losses are computed over all 12 displayed leads, the convention affects training as well as analysis. The low-rank factorization and exact lead relations constrain the decoder to respect ECG sensor geometry while still learning the source-to-lead projection from data.

### 2.5. PhysEncoder as an Amortized Constrained Estimator

The PhysEncoder was a one-dimensional residual convolutional network [34] mapping the 12-lead median beat to the bounded parameter vector, $E_{\psi }:y\left(t\right)\mapsto \hat{\theta }$. It is an *amortized approximation to a constrained batch parameter estimator*: for each record, a bounded least-squares inverse could be solved through the decoder within the same θ bounds (the per-sample oracle of Section 2.6); the encoder is trained on the same objective with parameters shared across the dataset and gives a single feed-forward inverse that approximates the per-record optimum (Section 3.2 measures how closely). It is not a Luenberger observer or a Kalman filter with a convergence proof, so we use estimator rather than observer terminology. Whether the parameters can be stably recovered from a given lead set is an identifiability question, quantified in Section 2.8 and Section 2.9.

### 2.6. Training Objective

The model was trained with a composite objective,

(10) $$\begin{array}{cc} L= & \lambda_{\mathrm{rec}}L_{\mathrm{rec}}+\lambda_{\mathrm{slope}}L_{\mathrm{slope}}+\lambda_{\mathrm{QRS}}L_{\mathrm{QRS}}+\lambda_{\mathrm{ST}}L_{\mathrm{ST}}\\ \; & +\lambda_{T}L_{T}+\lambda_{\mathrm{phys}}L_{\mathrm{phys}}+\lambda_{H}L_{H}, \end{array}$$

with normalized reconstruction $L_{\mathrm{rec}}={∥y-\hat{y}∥}_{2}^{2}/\left({∥y∥}_{2}^{2}+\epsilon \right)$, a slope term $L_{\mathrm{slope}}={∥\Delta y-\Delta \hat{y}∥}_{2}^{2}$, and segment losses (in-window mean squared error) over fixed QRS, ST and T-wave windows placed relative to the R peak at nominally −60 to 100, 60 to 140 and 120 to 450 ms (on the 100 Hz grid the first sample of each falls outside its inclusive bound, so the effective windows begin at −50, 70 and 130 ms in training and evaluation alike); the windows are not adapted to the QRS offset, so in wide-QRS records the ST-window overlaps the terminal QRS. The physical regularizer $L_{\mathrm{phys}}$ combined a normal-prior pull-to-center on the unconstrained *z*, an ST-source sparsity term on $\alpha_{\mathrm{ST}}$, and boundary penalties; the prior was weighted to pull the depolarization and repolarization time constants, which otherwise tend to sit at their bounds, more strongly toward their centers (four-fold for $\tau_{\mathrm{dep}}$ and six-fold for $\tau_{\mathrm{rep}}$), which keeps these directions away from their bounds without loss of reconstruction quality. The lead-field regularizer was $L_{H}=\bar{H^{2}}+\lambda_{\mathrm{smooth}}\;\bar{{\left(H_{V_{k+1}}-H_{V_{k}}\right)}^{2}}$, where $H_{V_{k}}$ is the row of $H_{\mathrm{ind}}$ that projects the sources onto precordial lead V*k* (k=1,…,6), the overbars denote entry-wise means and $\lambda_{\mathrm{smooth}}=1$; the second term asks adjacent precordial leads to receive similar projections. Loss weights were $\lambda_{\mathrm{rec}}\;=\;1.0$, $\lambda_{\mathrm{slope}}\;=\;0.2$, $\lambda_{\mathrm{QRS}}\;=\;1.0$, $\lambda_{\mathrm{ST}}\;=\;0.5$, $\lambda_{T}\;=\;0.5$, $\lambda_{\mathrm{phys}}\;=\;0.05$, and $\lambda_{H}\;=\;0.001$ (Table S3, Supplementary Materials). A per-sample oracle fit on the same objective establishes the decoder’s expressive capacity, the ceiling any amortized inverse can reach; the encoder is trained on the same objective over the full training fold. The oracle estimates are a reference, not a training target.

### 2.7. Interpretable Descriptors and Classifiers

From $\hat{\theta }$ and the reconstructed ECG, we computed disease-oriented descriptors. For conduction, we used the QRS-duration measured on the reconstructed waveform, which tracks the model-independent QRS-duration of the real beat and is stable across training seeds (Section 3.6); an internal proximal-delay descriptor was found to be seed-unstable and is reported only as a negative example. Repolarization features included node-level APD/recovery descriptors and ECG-domain T-wave features (T-peak amplitude, area, sign, and cross-lead T-wave sign discordance). ST features included internal source norms and lead-projected descriptors ${\mathrm{ST}}_{\mathrm{lead}}=H_{\mathrm{ind}}\alpha_{\mathrm{ST}}$. The primary classifier was XGBoost [33]; logistic regression was run as a supplementary robustness check. The auxiliary recovery descriptor is a surrogate-stability proxy: the training-fold *z*-scored sum of four dispersion descriptors (activation, T-end, repolarization time constant, projected-ST magnitude) and its negative, two collinear columns adding one direction; the finite-time recovery multiplier of Section 2.10 is computed on a small subset only and enters no feature group. Feature groups were C5 (the main interpretable model; θ + repolarization + projected ST + auxiliary recovery descriptor, of which only the auxiliary recovery descriptor is non-interpreted and is kept for ablation completeness), C2 (θ + repolarization), and C6 (a compact heuristic subset of 31 descriptors, assembled by hand rather than from the Fisher ranking); the additional ablation groups (C0, C3, C4) are defined in Section 3.4. Folds 1–8 were used for classifier fitting and feature scaling, fold 9 for any selection that a decision rule requires, and fold 10 for evaluation only.

### 2.8. Fisher Information Identifiability and Lead-Set Reduction

To quantify which parameters can be stably inferred from a given lead set S, we computed for each record *n* a local sensitivity matrix $S_{n}=\left(\partial \mathrm{vec}({\hat{y}}_{S})/\partial \theta \right)\;R$ by forward-mode automatic differentiation at the encoder estimate ${\hat{\theta }}_{n}$, where ${\hat{y}}_{S}$ stacks the leads of S over the *T* samples and $R=\mathrm{diag}(\theta_{\mathrm{radius}})$ scales each column by the parameter’s box radius (Equation (6)). The decoder and lead-field are held fixed, so every statement below is conditional on the learned decoder and concerns θ alone. Under additive Gaussian output noise with covariance Σ the Fisher information of one record is $S_{n}^{⊤}\Sigma^{-1}S_{n}$, which we average over a design subset of *N* records,

(11) $$F\left(S\right)=\frac{1}{N}\sum_{n=1}^{N}S_{n}^{⊤}\Sigma^{-1}S_{n},$$

a population-level design criterion for the lead set rather than the bound for any single patient. This is a *local* identifiability analysis: *F* reflects the local geometric sensitivity of the reconstructed leads to the equivalent parameters at the operating point, not unique recovery of true anatomical or ionic parameters [39]. The primary analysis uses an identity-noise model, Σ=I (the Gauss–Newton approximation). Real ECG noise is colored and lead-dependent, and a non-identity Σ down-weights noisier leads, so the identity-noise optimum should be read as an idealized, noise-agnostic result. To quantify its sensitivity, we repeated the selection under an empirical diagonal Σ whose per-lead variances are the DC-removed variances of the pre-QRS segment of the training-fold median beats (−300 to −110 ms; Table S17). That segment contains baseline noise and the atrial deflection the surrogate does not model, so Σ is a data-derived lead weighting by unmodeled pre-QRS variance rather than a calibrated electrode-noise model ($\Sigma^{-1}$ is normalized to unit mean over the leads so that the ridge below is comparable across the two weightings); we retain the identity-noise result as the primary reference because median beats have already undergone substantial noise reduction; what changes and what survives under the empirical weighting is reported in Section 3.7. For a given lead set, ${\hat{y}}_{S}$ contains every displayed lead of the set, so the 12-lead FIM includes the four derived limb leads through their exact relations (Section 2.4); under Σ=I they count as additional observations although they are not independent measurements (Section 3.7 also gives the eight-independent-lead value); the lead *selection* of Section 2.9, which needs additivity over leads, is restricted to the eight independent leads. The spectral-entropy effective rank [40,41] of *F* was

(12) $$r_{\mathrm{eff}}=\exp\;\left(-\sum_{i}p_{i}\log p_{i}\right),\;p_{i}=\frac{\nu_{i}}{\sum_{j}\nu_{j}},$$

with $\nu_{i}$ the eigenvalues of *F*. The lead-set *selection* of Section 2.9 is made on a 400-record design subset of the validation fold 9 (100 per class, drawn with the run’s seed) and is identical when the subset is drawn from the training folds 1–8 (Table S18); the 400-record fold-10 subset on which Section 3.7 reports effective ranks, Cramér–Rao bounds and log det margins serves only to evaluate the selected and the reference lead sets, so no lead set is chosen on the test fold. The five-seed effective ranks evaluate all five checkpoints on the locked model’s fold-10 subset, and the identifiability tiers are unchanged when the per-parameter scores are recomputed that way (Table S11). We evaluated the full 12 leads and the reduced sets II+V1+V5, I+II+V5, and lead II alone; II+V1+V5 is the set we call the conventional clinical set: V1 and II are the pair the AHA practice standards recommend for continuous arrhythmia monitoring [42,43] (V1 for QRS morphology, II for P-wave visibility), and V5 adds lateral coverage.

Per parameter we report an *identifiability score*

(13) $$s_{i}={\left({\left[{\left(F+\lambda I\right)}^{-1}\right]}_{ii}\right)}^{-1/2},\;\lambda =10^{-3},$$

the reciprocal of the Cramér–Rao standard deviation of $\theta_{i}$. Without the ridge, ${\left[F^{-1}\right]}_{ii}$ is the classical local Cramér–Rao bound on the variance of an unbiased estimate of $\theta_{i}$; every Cramér–Rao bound reported below is the ridge-regularized quantity, which keeps the inverse defined when *F* is near-singular, never exceeds the classical bound, and serves as an uncertainty proxy where *F* is rank-deficient. Because the Jacobian columns are scaled by each parameter’s box radius (Equation (6)), $s_{i}$ is dimensionless and comparable across parameters of different physical units: a score *s* corresponds to a one-standard-deviation uncertainty of 50/s percent of that parameter’s admissible range, so s=15 means ±3% of the range and s<1 means the posterior is wider than the range itself. We use this score to sort parameters into identifiability tiers in Section 3.7, requiring s≥15 in every seed for the strongly identifiable tier and s<1 in every seed for the primarily regularized tier.

### 2.9. Observability-Based Lead Selection

Beyond asking how identifiability depends on the number of leads, we posed reduced-lead *design* as an observability-based optimal sensor-selection problem: choose the lead subset S that maximizes the identifiability of the mechanistic parameters [44,45]. Because the per-lead Fisher contributions are additive, $F\left(S\right)=\sum_{l\in S}F_{l}$ (verified numerically), selection was restricted to the eight *independent* leads {I, II, V1–V6} that the decoder predicts directly; the augmented and III leads are exact linear combinations of I and II and are therefore not independent measurements (under the exact relations their noise covariance is singular), so they cannot be counted as separate sensors. Recent reduced-lead reconstruction work likewise operates on these eight independent leads [18]. Because the three-lead FIMs are ill-conditioned, the criteria are evaluated on the ridge-regularized matrix $F_{\lambda }\left(S\right)=F\left(S\right)+\lambda I$ with the same $\lambda =10^{-3}$ as in Equation (13). We evaluated the three standard scalar criteria [44],

(14) $$\begin{array}{cc} D\text{-}\mathrm{optimal}:\; & \max_{S}\log\det F_{\lambda }\left(S\right),\\ A\text{-}\mathrm{optimal}:\; & \min_{S}\mathrm{tr}F_{\lambda }{\left(S\right)}^{-1},\\ E\text{-}\mathrm{optimal}:\; & \max_{S}\nu_{\min}\;\left(F_{\lambda }\left(S\right)\right), \end{array}$$

solved by exhaustive search over the small subset space and, for the log det objective, also by greedy selection. Only the log det objective is submodular and monotone (for λ>0 it is the log-determinant with a prior precision λI), which gives greedy selection a (1−1/e) worst-case optimality guarantee [45,46,47]. We additionally reported per-parameter Cramér–Rao bounds $\sqrt{\mathrm{diag}F_{\lambda }^{-1}}$ and a singular-value decomposition of the source-to-lead operator H=AB to relate poorly identifiable parameter directions to low-gain (near-null) directions of the lead-field.

#### Reduced-Lead Encoders and the Per-Record Oracle

The reduced-lead classification of Section 3.7 uses one encoder per lead set: it receives only the leads of S as input, the decoder and lead-field stay frozen, and it is trained on the objective of Section 2.6 evaluated on all 12 reconstructed leads (the full-12 teacher), so that the unobserved leads are inferred through the fixed decoder; Table S2 reports the same encoders trained with the objective restricted to the observed leads. The per-record oracle of Section 2.6 is the second stage of the same estimator: with the decoder fixed, the unbounded *z* of one record is optimized from the parameter center by Adam until the median NRMSE stops improving, which gives the capacity reference of Table S14 and the alternative inverse of Section 3.7.

### 2.10. Auxiliary Finite-Time Recovery Descriptor

As an auxiliary control-theoretic check, surrogate parameters were mapped to an eight-node Aliev–Panfilov-style paced AP-ODE with graph diffusion to obtain a finite-time recovery multiplier. Because the paced trajectories did not converge to a periodic orbit, it is *not* a Floquet multiplier; the full analysis is reported in the Supplementary Materials only (Figure S1, Table S1).

### 2.11. Evaluation and Statistical Analysis

Reconstruction was evaluated by normalized root-mean-square error (NRMSE) overall and in the QRS, ST, and T windows of Section 2.6 and the P-window (−300 to −100 ms), Pearson correlation, ST mean absolute error in physical millivolts, and the parameter boundary rate. Classification was evaluated on fold 10 by macro-AUROC, macro-AUPRC, macro-F1, and balanced accuracy; macro metrics were computed one-vs-rest and averaged unweighted across the four classes, so each class contributes equally despite the test-set imbalance (NORM 912 vs. CD 184). Hyperparameters, loss weights, and parameter bounds were fixed using folds 1–8 (training) and fold 9 (validation) only; fold 10 was held out until all model and feature choices were locked, so no test-set information informed model or feature selection. Where a decision rule requires an operating point (Section 3.5), the threshold is selected on fold 9 and applied unchanged to fold 10, and the three-lead selection of Section 2.8 is likewise made on fold 9; fold 10 is scored, never searched over. To establish stability, the full training and evaluation pipeline was repeated across five random seeds; we report the locked seed-42 model together with the mean ± standard deviation (SD) across seeds and 95% patient-clustered bootstrap confidence intervals on test predictions (patients resampled with replacement; 1000 resamples). Seed 42 was fixed in advance as the first random seed (not selected for performance). Pairwise contrasts between feature groups use a paired patient-clustered bootstrap of the macro-AUROC difference (2000 replicates; Table S16) with two-sided *p*-values; group differences in interpretable features were assessed with Kruskal–Wallis tests, with Cliff’s δ as effect size. All multiple comparisons use the Benjamini–Hochberg false-discovery-rate (FDR) correction. Two intervals resample records instead: the lead-selection margin (200 resamples of the 400-record design subset) and the external cohorts (1000 resamples; no patient identifier is available).

## 3. Results

### 3.1. Reporting Convention for Seeds

The pipeline was trained and evaluated under five random seeds {42,1,2,3,4}; seed 42 was fixed in advance and is the *locked* model, from which every figure, confusion matrix, and per-record quantity is computed. Scalar summaries give the locked value first and the five-seed mean ± standard deviation (ddof =1) in parentheses; figure captions state which convention the panel uses. The locked seed is the more conservative choice for reconstruction and effective rank but not for the MI→NORM count (97 against 101.0±4.9) or the C5 macro-AUROC (0.9014 against 0.9001±0.0030).

### 3.2. ECG Reconstruction

**Figure 2:**
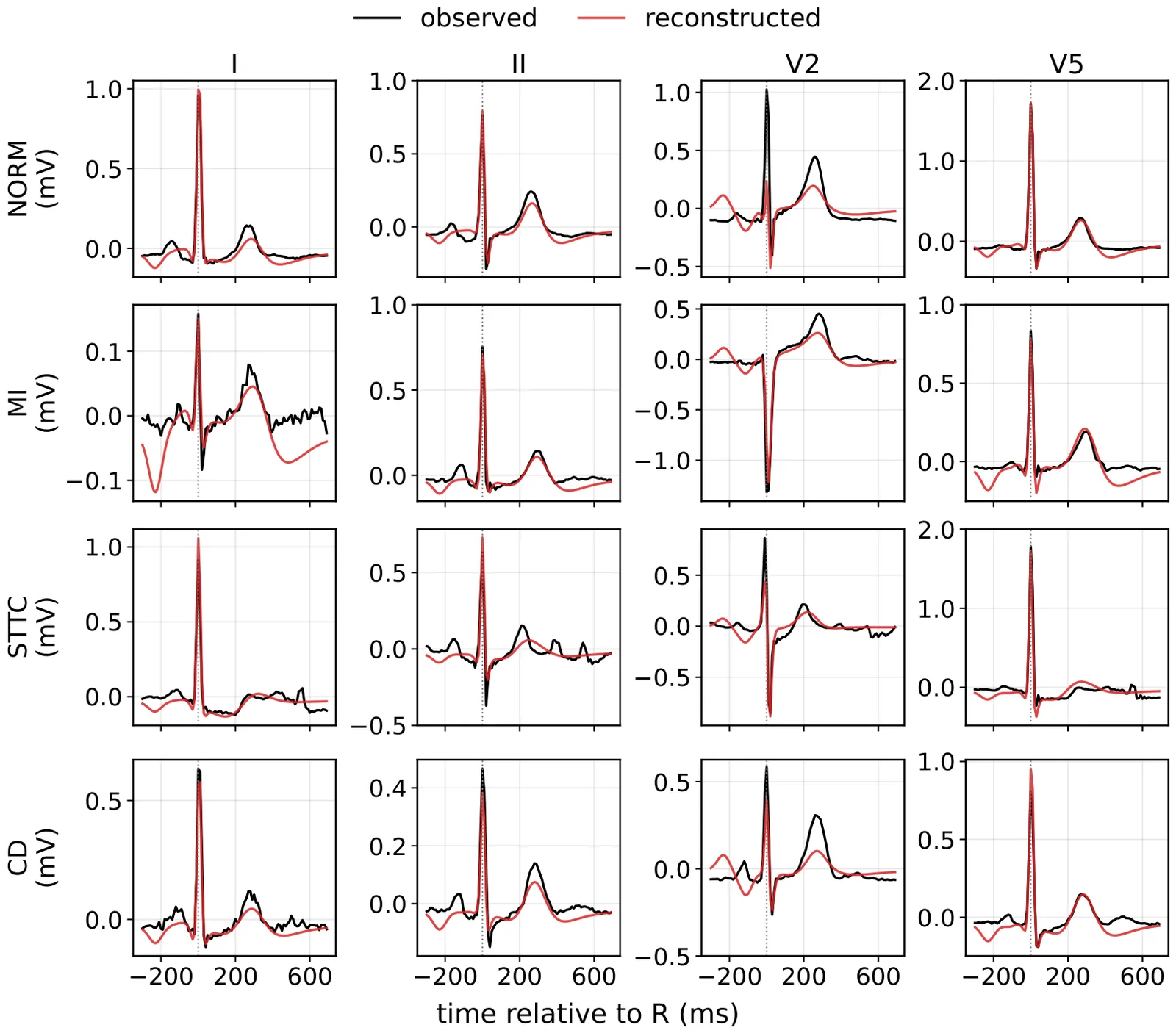
Representative reconstructions. Observed (black) versus reconstructed (red) median beats in leads I, II, V2 and V5 for one test record per class (NORM, MI, STTC, CD). The model captures beat morphology (overall median correlation 0.91; a full 12-lead example in Figure S2); absolute-amplitude detail (deep S waves, some T-wave peaks) remains approximate, consistent with the scaled NRMSE ≈0.40; frontal-lead reconstruction is weaker than precordial (lead I, left column), and the P-wave is not reconstructed (Section 3.2). The dotted vertical line in each panel marks the R peak (t=0).

The amortized PhysEncoder was stable across five training seeds on the test fold: scaled median NRMSE 0.401 (0.383±0.010), median correlation 0.912 (0.918±0.004), QRS-window NRMSE 0.244 (0.240±0.007), and ST mean absolute error 0.0143 mV (0.0147±0.0011) (Table 2). The parameter boundary rate was 0.127 (0.144±0.023); the most boundary-prone group was the internal ST-source $\alpha_{\mathrm{ST}}$, consistent with its low identifiability (Section 3.7). Figure 2 shows representative reconstructions, and Figure S2 shows a full 12-lead example. We report correlation alongside NRMSE because correlation is amplitude-insensitive: the model captures morphology (r≈0.91) while absolute amplitude remains approximate (scaled NRMSE ≈0.40). Reconstruction quality is spatially non-uniform: the anterior–lateral precordial leads (V3–V6, median per-lead correlation up to 0.95) are reconstructed most accurately, and the inferior limb-lead aVF least (0.89, the worst lead in all five seeds); the four derived limb leads reach 0.90–0.94, comparable to the directly predicted leads. The P-wave is not reconstructed. In a −300 to −100 ms window, the median NRMSE is 1.628 (1.30±0.24 across seeds) and the windowed correlation is 0.11, changing sign between seeds: no term of the objective supervises the atrial region, whose amplitude is about 14% of the QRS and contributes almost no gradient to the global reconstruction term. Adding an atrial-window term does not change this (Table S15): at weights of 0.5 and 2.0 the window NRMSE falls only to 0.96 and 0.88, the encoder silences the atrial source instead of fitting the P-wave, the atrioventricular delay stays constant across records, and the T-window error rises. No descriptor used in this paper is computed from the P-window, and the atrial parameters are not interpreted; the surrogate is a ventricular-morphology model.

Per-record fits on the same 1594 fold-10 records separate the encoder’s share of the residual from the decoder’s (Table S14). With the locked decoder held fixed, optimizing the parameters record by record reaches a median scaled NRMSE of 0.390 against the encoder’s 0.401, so the amortized PhysEncoder lies within about 0.011 NRMSE of the per-record optimum of its own decoder (the per-record fits are early-stopped, so this gap is a lower bound). Refitting the shared lead field together with the per-record parameters reaches 0.295, and a lead field refit on one half of the fold transfers to the other half (0.312 against 0.393 with the locked lead field); about 0.1 NRMSE of the residual is therefore attributable to the lead field learned under amortized training, not to the parameterization or the encoder (Section 4.5).

### 3.3. Propagation of Reconstruction Error to the Descriptors

Because the descriptors are read off the reconstruction, reconstruction error could in principle contaminate them. Two controlled tests bound how much it does.

First, a substitution oracle. Six C5 descriptors are computed from the reconstruction and have an exact observation-domain twin; replacing all six with their twins raises macro-AUROC by 0.0042±0.0007 across seeds (positive in 5/5), and the gain falls entirely on ST/T-change (recall 0.638→0.679) while MI is untouched (0.476→0.473), so reconstruction fidelity does not explain the MI deficit. Descriptor fidelity itself splits by kind (Table S12): of the 37 reconstruction-domain descriptors with an observed twin, 21 of the 24 amplitude, area and slope descriptors recover with r≥0.80 (the three exceptions are the low-amplitude Tlowamp family), all 12 T-wave timing and shape descriptors fall below 0.50, and the single polarity descriptor, the reconstruction-domain T-wave sign discordance used in Section 3.6, reaches only r=0.37±0.12; it is one of the six descriptors substituted above, so its MI association is a model-domain quantity rather than a proxy for the observed discordance.

Second, stratification by reconstruction quality. Within class, MI recall *rises* from 0.375 in the best-reconstructed NRMSE quartile to 0.634 in the worst (Δ=+0.259±0.065, 5/5 seeds), and 67% of all MI→NORM errors sit in the two best-reconstructed MI quartiles (Table S12). An infarction whose morphology stays close to normal is both easy to reconstruct and easy to call NORM, whereas a grossly abnormal beat reconstructs poorly and is correctly flagged: reconstruction error and detectability are coupled through distance from normal morphology, so a low reconstruction error does not indicate that the disease call is reliable. The reconstruction self-check of Section 4.4 flags model misfit, not diagnostic confidence.

### 3.4. Disease Prediction from Interpretable Descriptors

Classification accuracy is not the contribution of this work; the numbers below are to be read against the black-box reference (Section 4.1).

**Figure 3:**
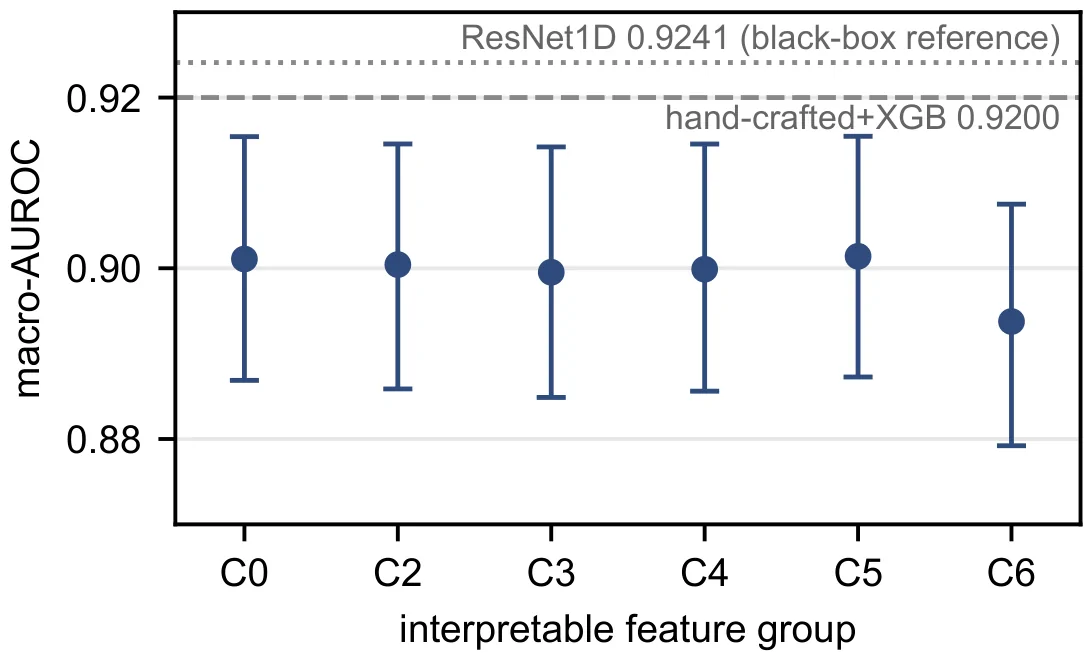
Classification performance of the interpretable feature groups (fold-10 test, seed-42; points are macro-AUROC, error bars 95% bootstrap CIs). The five θ-based variants (C0, C2–C5) cluster near 0.90; the reduced 31-descriptor set C6 sits below them. Dashed/dotted lines mark the black-box reference (hand-crafted + XGBoost 0.9200, ResNet1D 0.9241). The *y*-axis is truncated to 0.87–0.93 for visualization only; error bars are marginal CIs, not a pairwise test (Section 3.4).

**Figure 4:**
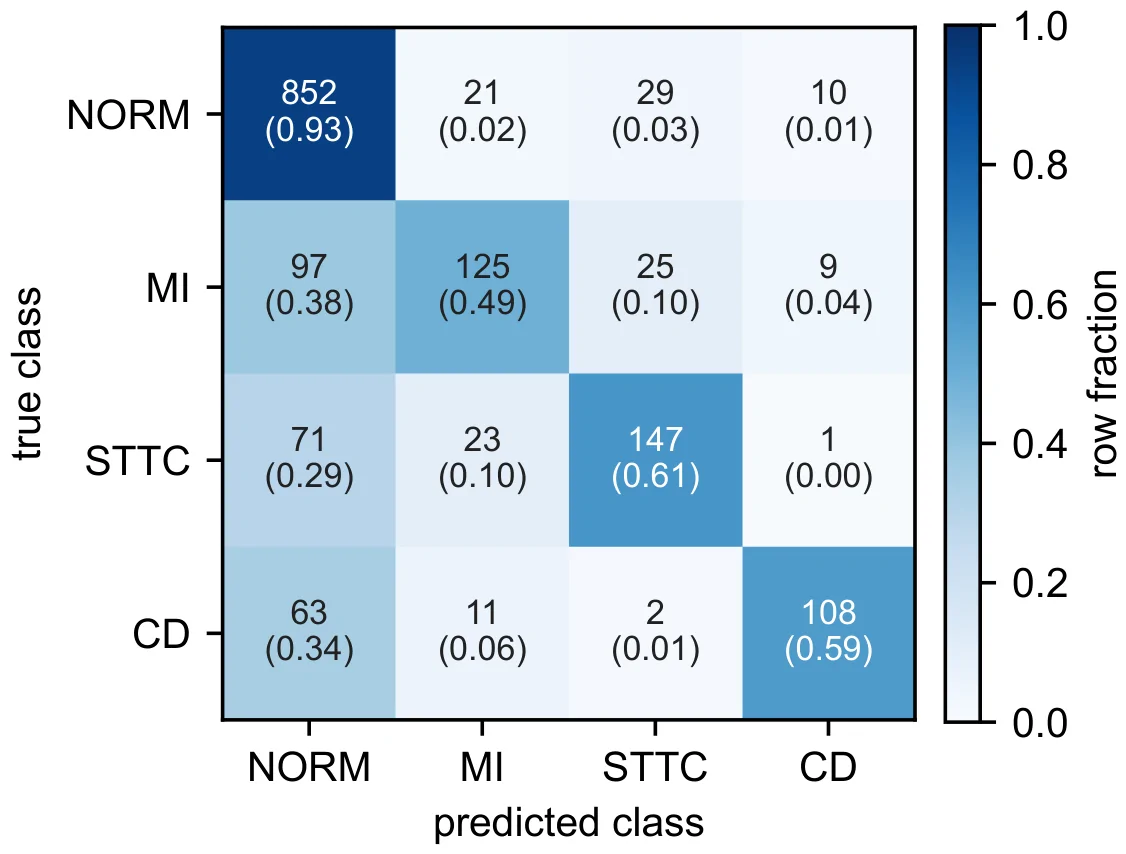
Per-class confusion matrix of the main interpretable model at the argmax operating point (C5, seed-42, fold-10 test); rows are true classes, normalized to sum to one.

Black-box baselines established a reference near macro-AUROC 0.92: hand-crafted XGBoost reached 0.9200 and ResNet1D reached 0.9241 (Table 1). The interpretable models approached this reference while using only surrogate descriptors. Across five seeds, the main interpretable model (C5) reached a macro-AUROC of 0.9014 (0.900±0.003) and the parameter-only model (C0) 0.9011 (0.901±0.003) (Table 3). Across the five θ-based groups the seed-42 macro-AUROC spans 0.8995–0.9014 and the five-seed means span 0.8990–0.9012 (per-group standard deviation ≈0.003). A paired patient-clustered bootstrap over all 15 pairwise contrasts, with the Benjamini–Hochberg correction used throughout (Section 2.11), finds no significant difference among these five groups; the smallest uncorrected *p* is 0.11. C5 is the main model because it alone carries the full set of *interpreted* descriptors (QRS-duration, repolarization and T-wave morphology, lead-projected ST). The auxiliary recovery descriptor contributes negligibly to classification (C4 vs. C0; Table 3). The one group that does separate is C6, the reduced 31-descriptor compact heuristic set, which falls below C5 by 0.0076 (95% CI [0.0026,0.0128]; all 15 contrasts in Table S16). Compressing 71 descriptors to 31 therefore costs a small but measurable amount of discriminative information. The interpretable variants sit ≈0.02–0.025 macro-AUROC below the black-box reference (Figure 3), and the per-class confusion matrix (Figure 4) shows MI as the most confused class. At the operating point, per-class sensitivity (recall) was uneven (NORM 0.93, STTC 0.61, CD 0.59, MI 0.49; balanced accuracy 0.654), so the macro-AUROC near 0.90 should not be read as uniform clinical performance; in particular, MI, the clinically critical class, is under-detected. Table S4 reports the full per-class metrics. Of the 256 MI test records, 97 (about 38%) were assigned to NORM, 25 to STTC, and 9 to CD; MI predictions were comparatively precise (precision 0.69) but recall was low (0.49). MI is also the weakest class for both black-box baselines on this subset (per-class AUROC 0.910 for ResNet1D and 0.908 for the hand-crafted XGBoost, against 0.909–0.945 for the other classes), as expected for a superclass that pools infarctions of any age and territory [30,37]; the surrogate’s own loss relative to ResNet1D is largest on MI (0.033 against 0.015–0.024 for the other classes). This concentration of MI→NORM errors is consistent with the lead-field analysis (Section 3.7): the internal ST-source $\alpha_{\mathrm{ST}}$ is the least observable direction, so the subtle, territory-dependent ST and Q-wave changes that separate many—especially older or non-anterior—infarctions from NORM are only weakly represented in the recoverable descriptor subspace. The evidence is associative: the substitution oracle and the decision-rule analysis exclude reconstruction fidelity and the operating point, so the deficit is most plausibly a property of what the descriptors resolve rather than of classifier capacity; the mechanism itself is not established here (Section 3.7 and Section 4.5).

#### Structural Ablation

Table 3 varies the descriptors fed to the classifier; Table S20 varies the surrogate itself, retraining one variant at a time with every other setting of the locked recipe unchanged (seed 42; training details in the caption). A rank-1 lead field cannot represent the data (median NRMSE 0.707, C5 macro-AUROC 0.856), whereas rank 5 reconstructs better than the locked rank 3 (0.341 against 0.401) at a classification inside the locked interval (0.897) with a 12-lead effective rank of 5.98, and the unconstrained rank 8 reaches 0.371 and 0.904; rank 3 is therefore a parsimony choice rather than a necessity. Removing the physics loss, the exact limb-lead relations, or the conduction graph leaves classification inside the locked model’s interval (C5 0.897, 0.901 and 0.894) and reconstruction as good or better, so none of the three is needed for accuracy. What each buys is physical consistency (last columns of Table S20): without the physics loss 30% of the parameters sit on their bounds (13% in the locked model); with a free 12-row lead field the reconstructed limb leads violate the Einthoven–Goldberger relations by 2% relative error (34% when the physical coefficients are applied in scaled coordinates, zero by construction in the locked model); and with free activation times some node activates before its parent on the conduction tree in 98% of test records, so the activation parameters that Section 3.6 interprets are physiological because the graph makes them so, not because the data do.

### 3.5. Decision Rules and the Cost of Raising MI Sensitivity

The recall values above are read at the argmax of the posterior, the only decision rule defined without a target sensitivity and hence the only one under which the ablation, the reduced-lead comparison and the external cohorts remain comparable. Because MI sensitivity is the clinically consequential number, we quantified how far it can be raised on the locked model and at what cost; thresholds were selected on fold 9 and applied unchanged to fold 10, and intervals are patient-clustered bootstrap intervals (Table S7).

Three mechanisms act separately. The class prior: the clean subset raises the NORM:MI ratio from 1.74 to 3.58, and rescaling the posterior to the full-PTB-XL frequencies lifts MI recall from 0.488 [0.421,0.559] to 0.559 [0.489,0.624]. Cost-sensitive fitting: inverse-frequency class weights give 0.570 [0.504,0.635]. An explicit MI threshold: at a fold-9 target of 0.90 the fold-10 recall is 0.922 [0.880,0.959] and MI→NORM errors fall from 97 to 16 of 256, at MI precision 0.295 and NORM recall 0.629—799 of 1594 records referred.

Macro-AUROC is essentially unmoved (0.9014, 0.9021, 0.8997): ranking capacity is a property of the representation, and re-deciding moves only the operating point. The reported recall thus has two separable sources—a representation-level ceiling, reflected in the lowest per-class AUROC (0.88; Table S4) and consistent with the low observability of the internal ST-source (Section 3.7), and the inclusion criterion and argmax rule, which set where on that curve the recall sits. The prior and the threshold recover sensitivity; they do not raise the ceiling.

### 3.6. Physiological Interpretation by Diagnostic Class

**Figure 5:**
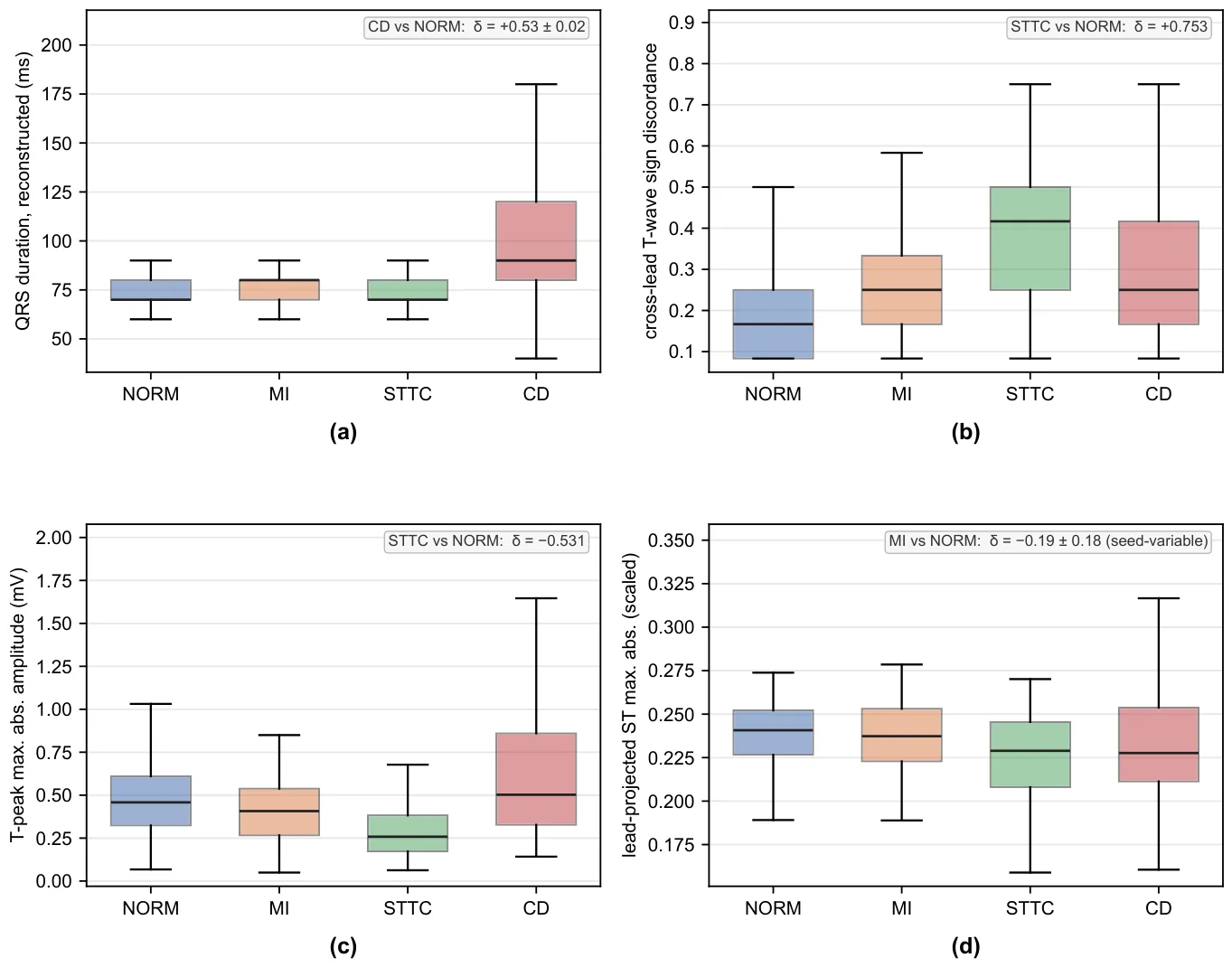
Core interpretable descriptors by diagnostic class (PTB-XL fold-10 test). (**a**) QRS-duration measured on the reconstructed waveform; higher in CD (Cliff’s δ=+0.53±0.02 across five seeds). (**b**) Cross-lead T-wave sign discordance, computed on the observed ECG; higher in STTC (δ=+0.753, identical across seeds). (**c**) T-peak maximum absolute amplitude, observed ECG; lower in STTC (δ=−0.531). (**d**) Lead-projected ST maximum absolute value, a parameter-derived quantity in the decoder’s scaled lead units; weak and seed-variable in MI (signed δ=−0.19±0.18), retained as the observability-limited negative example. Boxes show the interquartile range and median, whiskers extend to 1.5 × IQR, and points beyond the whiskers are not drawn. Cliff’s δ is versus NORM; the accompanying Kruskal–Wallis *p*-values are FDR-corrected (Section 2.11).

The inferred descriptors revealed disease-specific patterns consistent with standard ECG interpretation, and we report each effect as the mean±standard deviation of Cliff’s δ across five seeds so that the interpretation is shown to be seed-stable (Table 4, Figure 5).

*Conduction disturbance* was associated with increased QRS-duration measured on the reconstructed waveform (CD vs. NORM, Cliff’s δ=+0.53±0.02, positive in all five seeds; FDR $q<10^{-20}$). The reconstructed QRS-duration tracked the model-independent QRS-duration of the real beat (correlation 0.63; real-beat δ=+0.578), so the model recovers the same observable conduction marker that defines CD clinically. An internal proximal-delay descriptor, although appealing as a direct conduction-delay readout, was seed-unstable (its sign flipped across seeds) and is reported here only as a negative example, not as a claim-supporting quantity; this instability is itself consistent with the low identifiability of the proximal timing parameters (Section 3.7) and illustrates that descriptors closer to an observable quantity are more reliable.

*ST/T-change* was better characterized by directly observable T-wave morphology than by node-level APD dispersion: cross-lead T-wave sign discordance was strongly higher in STTC (Cliff’s δ=+0.753, identical across all five seeds because it is an ECG-domain signal feature independent of the model), and T-peak amplitude was lower (δ=−0.531), whereas node-level APD dispersion was *lower* in STTC. The latter runs opposite to the direction one would expect if the descriptor were merely by-construction, indicating that the class–descriptor associations are not tautological.

*Myocardial infarction* was most stably associated with T-wave morphology measured on the reconstruction: the reconstruction-domain T-wave sign discordance was higher in MI in all five seeds (δ=+0.24±0.05). The lead-projected ST descriptor, by contrast, was weak and seed-variable on this class (signed δ=−0.19±0.18, non-positive in all five seeds but with magnitudes ranging from 0.02 to 0.47), and its sign runs opposite the naive “ST-elevation” expectation; we therefore retain it as an observability-limited descriptor—consistent with the near-null internal ST-source identified in Section 3.7—rather than as a stable MI marker. MI remained the class with the largest seed-to-seed descriptor variation, consistent with its being the most challenging class (Section 4.5).

### 3.7. Lead-Set Identifiability and Observability-Based Lead Selection

We analyzed the surrogate parameters through the local Fisher information matrix (FIM) $F=S^{⊤}S$, with $S=\partial \mathrm{vec}(\hat{y})/\partial \theta$ evaluated at the encoder estimate $\hat{\theta }$ for each record and averaged over a fixed design subset of 400 fold-10 records (100 per class; Section 2.8).

#### 3.7.1. Monotonicity of Fisher Information Under Lead Removal

Because the Fisher information is additive over leads, $F\left(S\right)=\sum_{l\in S}F_{l}$ with each $F_{l}⪰0$, discarding a lead removes a positive-semidefinite term, so $F\left(S^{\prime }\right)⪯F\left(S\right)$ for $S^{\prime }\subset S$. Every Cramér–Rao bound is therefore non-decreasing, and log det *F* is non-increasing under lead removal, exactly and for every seed: a reduced lead set carries no more identifiable parameter information than the set it was drawn from, and generally less. This monotonicity is a property of the nested comparison and does not order two lead sets neither of which contains the other.

**Figure 6:**
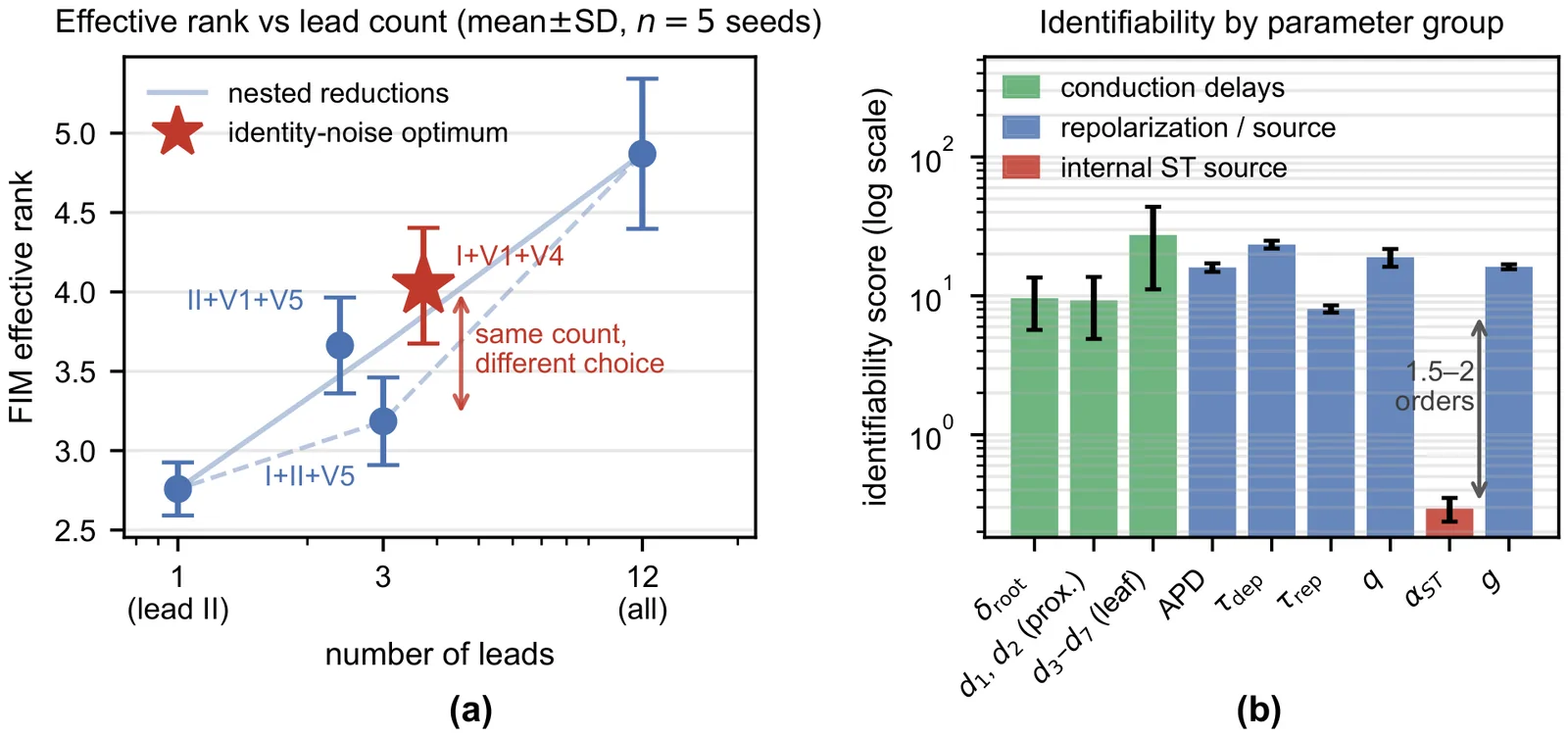
Lead-set identifiability: how much each lead set can recover (test subset, 400 records; error bars are the standard deviation across five training seeds). (**a**) Spectral-entropy effective rank of the Fisher information matrix against the number of leads; it measures spectral spread and is not itself monotone in lead count. The two faint lines are nested reduction chains (12 leads ⊃ a three-lead set ⊃ lead II); the three-lead sets, none of which contains another, are plotted side by side. The star marks the identity-noise optimum I+V1+V4, optimal under that noise model only (Section 3.7). The vertical spread at three leads shows how much lead *choice* matters at a fixed lead count; the 12-lead point uses all 12 displayed leads under Σ=I (Section 2.8). (**b**) Mean identifiability score by parameter group (log scale); the internal ST-source $\alpha_{\mathrm{ST}}$ is least identifiable by one and a half to two orders of magnitude.

The spectral-entropy effective rank of *F* summarizes this ordering: 4.362 (4.87±0.47) for the full 12 leads, 3.530 (3.66±0.30) for II+V1+V5, 2.840 (3.19±0.28) for I+II+V5 and 2.842 (2.76±0.17) for lead II alone (Figure 6a). It measures eigenvalue *spread*, not information content, and is not monotone in the number of leads (I+II+V5 scores marginally below lead II at seed 42); the ordering claims rest on log det *F* and the Cramér–Rao bounds, which are monotone by construction.

#### 3.7.2. Identifiability Tiers of the 47 Parameters

The 47 parameters are not equally constrained by the data. We tier them with the conservative per-parameter rule of Section 2.8 (Table 5; the full 47-row table is Table S11, which also records why a group-averaged criterion, admitting 25 parameters to the top tier, was rejected). Only 8 of 47 parameters are strongly identifiable—APD at nodes 3, 5 and 7, $\tau_{\mathrm{dep}}$ at nodes 3, 4, 5 and 7, and the global gain; *no* conduction delay and *no* ST-source amplitude reach that tier. All six $\alpha_{\mathrm{ST}}$ components fall in the regularized tier, with a one-standard-deviation uncertainty of 150–237% of their admissible range: the data do not constrain them, and their values are set by the prior. The near-null lead-field direction below gives the same conclusion independently. Only the eight Tier-A parameters and the descriptors derived from directly observable waveform features should be read as physiologically informative.

#### 3.7.3. Effective Dimension of the Identifiable Subspace

The bounded $\theta \in R^{47}$ spans the model’s *parameterization* space, of which the information spectrum at 12 leads has an effective rank of only about five, and fewer as leads are removed, so that the sensitivity of the leads to θ is concentrated in a low-dimensional subspace. We therefore treat θ as an *identifiability-aware* gray-box parameterization rather than 47 independently identifiable quantities and interpret only its high-observability, seed-stable directions (activation/QRS-duration and T-wave morphology). Consistently, the compact 31-descriptor set C6 retains most, though not all, of the discriminative information (Section 3.4), as expected if the diagnostic signal lives mainly in the observable subspace; C6 was assembled by hand rather than from the Fisher ranking, so this is a consistency observation, not a test. Free activation times do not raise these ranks (Table S20: 4.40 against 4.36 at 12 leads, 1.83 against 2.84 for lead II), because the graph’s coupling lets one lead constrain all of them (Supplementary Note S2).

#### 3.7.4. Do the Classifiers Use the Weakly Identifiable Directions?

Table S21 tests this on the fold-10 records. Displacing every parameter estimate along a random combination of the six least-informative directions of the population Fisher information by a quarter of the parameter radius changes the reconstruction by a median 0.0007 NRMSE, invisible in the data, yet flips 13% of the C5 decisions and lowers its macro-AUROC by 0.019; along the 24 least-informative directions at half a radius the reconstruction changes by 0.13 and 60% of the decisions flip, whereas the same step along the six most-informative directions destroys the reconstruction (0.99) while flipping 35%. An alternative inverse makes the same point: the per-record refit with the decoder fixed (Section 2.6) reconstructs fold 10 slightly better than the encoder (median NRMSE 0.390 against 0.401), reproduces the Tier-A parameters (median Pearson r=0.72 across records) but not Tiers B and C (0.35 and 0.41), and its features score 0.715 macro-AUROC under the encoder-trained C5 with 27% argmax agreement. The classifiers therefore use coordinates that the data do not pin down and that a different inverse sets differently: the reported accuracy is that of the encoder’s regularized inverse, a deterministic and smooth function of the waveform, not of the identifiable subspace alone. The physiological reading is therefore confined to Tier A and to directly observable waveform descriptors (Section 4.5).

#### 3.7.5. The Least Identifiable Direction Is the Internal ST-Source, and This Has a Lead-Field Origin

Across parameter groups, the leaf-edge conduction delays carry the highest five-seed mean score (27.9) but vary so much between seeds (3.4 at the locked seed) that none reaches the strongly identifiable tier, the proximal delays sit near the bottom of the identifiable range, and the internal ST-source amplitudes $\alpha_{\mathrm{ST}}$ are least identifiable by one and a half to two orders of magnitude (group mean 0.29 against 8–28 for every other group; Figure 6b). This reflects the measurement geometry rather than a numerical artifact. The bottom of the 12-lead FIM spectrum is dominated by $\alpha_{\mathrm{ST}}$: the five smallest eigenvalues span an effectively unobservable subspace whose energy is concentrated on the ST-source group (group energy 0.89 at the locked seed; 0.85±0.09 across the five seeds). Equivalently, the singular-value decomposition of the source-to-lead operator H=AB yields singular values [1.398,0.966,0.505,0,…] (rank three by construction), and the spatially uniform ventricular ST-source mode is mapped to the leads with a transmission gain of only 0.342 (0.40±0.15 across seeds), about one quarter of the leading mode and below the smallest nonzero singular value. The lead field thus has a low-gain (near-null) direction along the ST-source: changes in $\alpha_{\mathrm{ST}}$ produce almost no signal at the body surface and are therefore not recoverable from the standard leads. This near-null direction is a property of the rank-three, data-estimated lead-field of the surrogate rather than a claim about the true thoracic forward problem; a higher-rank or physically derived lead-field could render the spatially uniform ST mode more observable. The parameter scale contributes to the same effect: through this lead-field, an $\alpha_{\mathrm{ST}}$ component at its bound shifts a lead by only about 0.01 mV, an order of magnitude below the ST deviations in the data, so within its bounds the ST-source can neither be resolved nor express them ($\alpha_{\mathrm{ST}}$ and *q* are in arbitrary source units absorbed by the lead-field and the scaling). The leads observe the lead projection of $\alpha_{\mathrm{ST}}$, not the internal amplitude, and that projection is itself only a weak, seed-variable MI descriptor (Section 3.6); no ST-source quantity, internal or projected, is therefore claimed as an MI marker.

#### 3.7.6. Per-Parameter Cramér–Rao Bounds Under Lead Reduction

The Cramér–Rao bound (CRB, $\sqrt{\mathrm{diag}(F_{\lambda }^{-1})}$ in scaled units, the regularized quantity of Section 2.8) quantifies the cost of lead reduction. The CRB of $\alpha_{\mathrm{ST}}$—the worst-recoverable group—degraded monotonically from 3.39 at 12 leads (4.21 on the eight independent leads alone; under Σ=I the four derived leads count as additional observations of I and II) to 7.05 (II+V1+V5), 8.30 (I+II+V5), and 22.76 for lead II alone (Table 6). The internal ST-source thus becomes progressively unrecoverable as leads are removed.

#### 3.7.7. Observability-Based Lead Selection

The ordering above quantifies how identifiability depends on the *number* of leads; we next asked which *specific* leads a wearable system should retain, selecting among the eight independent leads under the D-, A-, and E-optimal criteria of Section 2.9.

**Figure 7:**
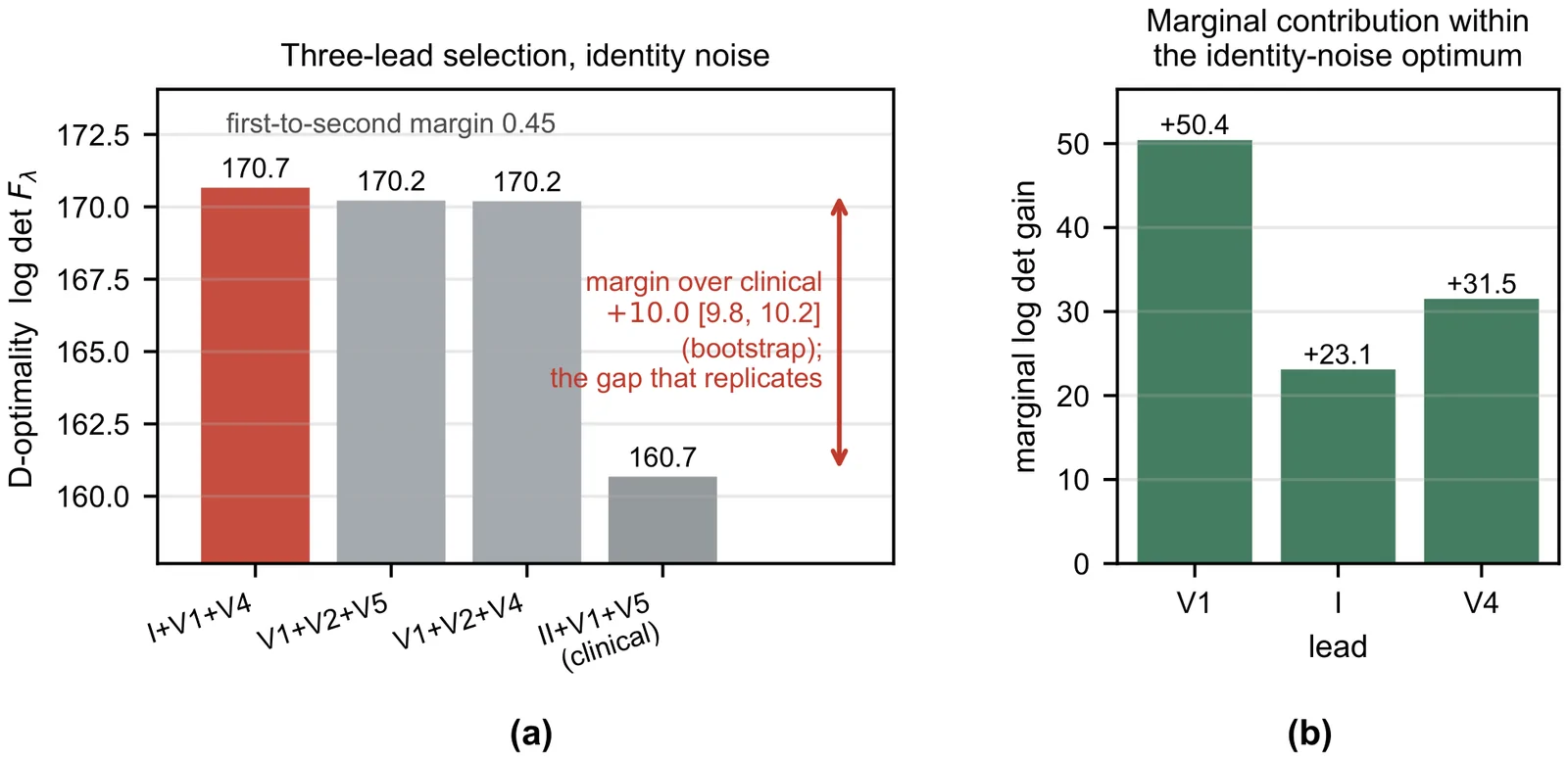
Observability-based lead selection: which leads to keep (test subset, 400 records, seed-42; identity-noise model throughout—under the empirical-noise covariance the optimum is a different triple, Section 3.7). (**a**) Three-lead selection under identity noise: $\log\det F_{\lambda }$ of the three best sets from an exhaustive search over all 56 subsets of the eight independent leads, with the clinical set II+V1+V5 shown for scale. Error bars are omitted because the bootstrap resamples records jointly for every set; the defined uncertainty is that of the paired difference against the clinical set (+9.98 point estimate; bootstrap mean +10.00, 95% CI [9.81,10.21]; +32.38 under the empirical weighting). The 0.45 margin over the second-ranked set is of the order of its between-seed variation, which is why the third lead does not reproduce across training seeds. (**b**) Marginal log det contribution of each lead in the selected set; large and comparable contributions indicate low redundancy and spatial complementarity.

For three leads, the D- and A-optimal criteria agreed on the same set, I+V1+V4, while the E-optimal criterion selected V1+V2+V5 (Figure 7a); the D-optimal set was also selected within each diagnostic class (NORM, MI, STTC, CD) separately, indicating that the optimal configuration is a property of the model and lead-field geometry rather than of any one class. The selected set differs from the clinical reduced set II+V1+V5 and scores higher on the D-optimal criterion: its D-optimal score $\log\det F_{\lambda }$ was 170.66 versus 160.68, a bootstrap-mean margin of +10.00 over this *clinical* reduced set (95% bootstrap CI [9.81,10.21]; the difference of the two point estimates is 9.98), the same three leads were selected in 100% of bootstrap resamples, and the margin remained 10.06 after flooring near-zero FIM eigenvalues, confirming that it is not driven by noisy near-unobservable directions. The margin over the second-ranked *observability* set, by contrast, was small (0.45 at seed 42; 0.26–1.48 across the five seeds), of the same order as its variation between training seeds; the third lead, unlike the gap to the clinical set, therefore, does not reproduce across training runs. The marginal log det contributions of the three leads were large and comparable (V1 +50.4, V4 +31.5, I +23.1; Figure 7b), indicating low redundancy.

Across the five seeds, under this identity-noise model, the I+V1 core was always selected while the third lead alternated between V4 (two seeds) and V5 (three). The design fold does not drive this result: the montage is the one the validation fold selects. With the 400-record subset drawn from fold 9 the D- and A-optimal set at seed 42 is again I+V1+V4 (margin 10.01, clinical rank 22), as it is on the training folds 1–8; across seeds the D- and A-optimal sets agree fold for fold, the margin over II+V1+V5 moves by at most 0.21 log det units and the clinical set’s rank by at most two places; only the E-optimal set, set by the smallest, ridge-dominated eigenvalue, changes (Table S18). The fold-10 values above are therefore an evaluation of a montage chosen elsewhere, not a search over the test fold.

#### 3.7.8. Sensitivity of the Selection to the Noise Model

Repeating the identical protocol—exhaustive search over all 56 three-lead subsets, bootstrap resampling, five seeds—under the empirical lead weighting ($W=\Sigma^{-1}$, Section 2.8) changes the answer: the optima become V2+V3+V5, V2+V4+V5, I+V2+V4, V1+V2+V4 and V2+V4+V5 (seed 42 first; its optimum shares no lead with I+V1+V4), so neither I nor V1 is a stable member. The specific montage is therefore a property of the assumed noise model, and we do not present one. Counting selected leads cannot establish precordial dominance, because of the base rate: the selection pool holds two limb and six precordial leads, so a random triple contains 2.25 precordial leads on average and is all-precordial with probability 20/56=0.36. The identity-noise optima average 2.0 precordial leads (below chance) and the empirical-noise optima 2.8 (above it), although with five seeds that share one 400-record subset this excess cannot be tested formally.

Retraining the two named montages without the full-12 teacher loss moves their macro-AUROC by +0.007 and −0.001, well within the width of the patient-clustered intervals, and the largest optimism in any matched comparison is 0.011 (Table S2), so teacher supervision does not account for the reduced-lead performance reported here. The marginal intervals of Table 6 are not a pairwise test, so Table S23 compares the four lead-set models by the paired patient-clustered bootstrap of Section 3.4: five of the six contrasts are significant after Benjamini–Hochberg control, the 12-lead model exceeding the clinical set by 0.0143 [+0.0056,+0.0231] and the observability-optimal set by 0.0233 [+0.0140,+0.0324], and both three-lead sets exceeding lead II alone by 0.0428 and 0.0338; the one contrast that is not significant is the observability-optimal set against the clinical set (−0.0090 [−0.0198,+0.0012], p=0.092), so the two montages are not distinguishable on this endpoint at this sample size, which is a weaker statement than equivalence. All these results nonetheless come from simulated lead removal on 12-lead records, not from devices that record three leads (Section 4.5).

Three conclusions do survive both noise models. First, lead II is never selected: under either weighting it belongs to the optimum at no seed; at the locked seed it appears in no top-five set, and the conventional reduced set II+V1+V5 ranks 22nd of 56 under identity noise and 48th of 56 under the empirical-noise model (never better than 5th or 34th at any seed). Second, every optimum contains at least one of the precordial leads V2–V5, the leads with the least unmodeled variance relative to their own signal scale (Table S17). Third, the gap between the observability-optimized set and the clinical set is large under both weightings (+10.00 and +32.38 log det units at seed 42), so the conclusion that conventional reduced-lead placement is not observability-optimal is noise-model-invariant even though the replacement montage is not. The precordial advantage is a signal-to-variance effect, not a quieter electrode: in millivolts the pre-QRS variance is comparable across all 12 leads, whereas relative to each lead’s own scale, which is what the Fisher information weights, V2–V5 carry a 1.5–2.1× smaller unmodeled pre-QRS standard deviation than the limb leads because their signal amplitude is larger (Table S17). Placed on the degradation curve (Figure 6a), the optimal three-lead set attains an effective rank of 3.72 (4.04±0.36) versus 3.66±0.30 for the clinical II+V1+V5. I+V1+V4 is optimal for mechanistic-parameter observability in this surrogate, not a clinical recommendation; the conventional leads serve other purposes (lead II for rhythm and P-wave visibility, which the surrogate does not represent and the criterion therefore cannot value; V5 for lateral ischemia).

#### 3.7.9. Further Noise Models and Wearable-Feasible Families

Two noise models cannot establish how stable the selection is, so Table S22 repeats the search under five more, all expressed through Σ of Section 2.8: the full pre-QRS lead covariance of the training folds (off-diagonal lead correlations up to 0.88), that covariance combined with AR(1) temporal correlation (ρ=0.8), AR(1) correlation alone, and two artifact stress tests that quadruple the noise variance of the limb leads or of the precordial leads. The montage moves with the model (9 distinct optima across seven models and five seeds), but the ordering claims hold: II+V1+V5 is never better than 5th of 56 and its margin to the optimum is 6.4–32.3 log det units; lead II enters an optimum only when the precordial variance is quadrupled (I+II+V1 at every seed) and at one seed under the full-covariance–AR(1) model; and every optimum contains one of V2–V5 except under that precordial-artifact model. Under the full lead covariance, the optimum is I+V2+V4 at all five seeds. Wearable-feasible families make the value of precordial information concrete (seed 42, identity noise): a chest-only triple (V1+V2+V5) lies within 0.44 log det units of the unconstrained optimum, whereas a limb-based device allowed one chest electrode (best I+II+V1) loses 9.7 units and barely exceeds the clinical set (+0.29), and requiring lead II (II+V1+V4) costs 7.6 units (Table S22).

### 3.8. Single-Lead Analysis

With the standard lead-set-specific encoder (full-12 teacher), lead II reaches macro-AUROC 0.844 (Table 6); across sanity-check variants (encoder capacity halved, regularization increased, full-12 teacher removed), its descriptor-based AUROC remained in the range 0.841–0.848 (Table S2), so the single-lead performance is not a capacity or teacher-amortization artifact. However, lead II had the lowest five-seed-mean FIM effective rank (2.76±0.17) and the worst per-parameter Cramér–Rao values, and without full-12 teacher supervision its full-12 reconstruction degraded sharply. Lead II therefore contains nontrivial class-discriminative information in this clean four-class subset but does not provide stable recovery of multi-region cardiac descriptors.

### 3.9. Behavior Under Multi-Label Conditions

The main analysis uses a clean single-label subset so that identifiability is not confounded by overlapping diagnoses, at the cost of removing 6090 of 21,799 records. Applying the locked seed-42 encoder and decoder zero-shot (frozen scaler, nothing refitted) to the canonical PTB-XL superdiagnostic task (21,388 records with at least one superclass, official folds, five labels including the excluded hypertrophy class), reconstruction degrades modestly: median scaled NRMSE 0.4068 and correlation 0.9069 over the whole fold-10 pool (n=2158) against 0.4009 and 0.9115 on the clean subset, and 0.4440 and 0.8788 on the 508 records carrying more than one superclass (Table S13). With only the downstream classifier fitted on the multi-label training folds, five-label macro-AUROC is 0.8941 [0.8850,0.9023] from the 47 parameters alone, with MI at 0.8700 and hypertrophy, never trained for, at 0.8888. The descriptor space is thus not an artifact of the clean subset. The gap to the four-class value (0.9014) is under one point of macro-AUROC, but the two evaluations differ in label set, record set, and one-vs-rest negatives, so we do not read it as a measure of how much easier the clean task is. Multi-label *training* of the surrogate and a multi-label external cohort remain untested.

### 3.10. External Validation on Independent Cohorts

To test whether the findings are specific to PTB-XL, we applied the locked seed-42 model to two independent 12-lead cohorts with no retraining, fine-tuning, or rescaling (the PTB-XL training scalers were reused): the Georgia database [48] (United States; n=2586 of 10,344 after single-label filtering) and CPSC2018 [49] (China; n=4305). Records were resampled to 100 Hz and passed through the identical median-beat pipeline, and SNOMED-CT codes were mapped to the four superclasses (Table S5). The external criterion is stricter than the internal one: SNOMED-CT carries no diagnostic-class attribute that would set rhythm, axis, and ectopy codes aside as PTB-XL’s form statements are, so a record was retained only if every code mapped to exactly one target superclass and none to hypertrophy. This criterion removed 75% of Georgia (61% for a rhythm, axis, or ectopy code, 13% for hypertrophy overlap, 1% for two target superclasses) and 37% of CPSC2018; Georgia retains 12% of its STTC-coded and 13% of its CD-coded records against all of NORM, so the external cohorts are rhythm-clean subsets (disposition and record manifests in Table S6).

Reconstruction transferred with little degradation: the median correlation was 0.887 (Georgia) and 0.873 (CPSC2018), versus 0.912 on PTB-XL (Table 7). Disease classification, evaluated with the parameter-only C0 model (matching the internal C0 reference; C0 and the main model C5 are statistically indistinguishable internally, Table 3), degraded: the macro-AUROC over the present three classes (NORM/STTC/CD) fell to 0.838 (95% CI [0.821,0.853]) on Georgia and 0.827 ([0.818,0.837]) on CPSC2018, from a class-matched internal value of 0.919 (the four-class internal C0 macro-AUROC is 0.901; per-class AUROC, AUPRC and $F_{1}$ with the record manifests in Table S6). The transfer pattern tracks observability: the most observable class—conduction, read from QRS timing—transferred well (recall 0.67 and 0.59, against 0.59 internally under the same classifier and decision rule), whereas the repolarization-driven ST/T-change transferred worst, and did so very differently in the two cohorts (recall 0.47 on Georgia, 0.27 on CPSC2018). The cohorts’ STTC labels differ in composition (Table S9): among clean STTC records CPSC2018 consists of its two ST-segment classes, ST-depression (81%) and ST-elevation (19%), with no T-wave-coded record; Georgia is dominated by nonspecific ST–T abnormality (65%) and T-wave codes (49%); and PTB-XL’s own superclass is T-wave dominated (55%) and contains neither an ST-depression nor an ST-elevation category, both being annotated as form statements. CPSC2018’s STTC pool therefore has essentially no counterpart in the superclass the model was trained on. The strongest STTC discriminator in the descriptor pool is the observed-ECG cross-lead T-wave sign discordance (Section 3.6; its reconstruction-domain twin tracks it only weakly), which the Georgia label set expresses and the CPSC2018 set does not, in the same order as the recalls; label heterogeneity and the low observability of the internal ST-source are not separable from these data alone. **MI could not be externally validated** because of the cohorts’ labeling, not because of our filter: only 7 of the 10,344 Georgia records carry the infarction code, each with a second target superclass, CPSC2018 contains none, and neither cohort annotates infarction territory or age (Table S9).

The observability analysis, by contrast, transferred to both cohorts (Table S19). The FIM effective rank decreased from the full 12-lead set to single-lead II in every cohort; the observability-optimal triple exceeded the clinical II+V1+V5 set in effective rank throughout (4.08 versus 3.65 on Georgia; 3.56 versus 3.17 on CPSC2018); and the greedy D-optimal three-lead set of the locked model replicated on both external cohorts (I+V1+V4 in all three datasets), so the optimum of a fixed trained model transferred across cohorts. Because within PTB-XL the third lead is seed-dependent and, under the empirical-noise covariance, neither I nor V1 is a stable member (Section 3.7), this replication shows that the selection procedure transfers under a fixed noise model (Section 2.8), not that I+V1+V4 is a universal triple. The lead-field singular spectrum, and hence the near-null ST direction, is a structural property of the learned decoder and is identical across cohorts by construction, so it is not independent cross-dataset evidence. The external check is therefore a transportability test of one trained model, not an independent validation of the selection mechanism, which would require a surrogate estimated on the external cohort itself (Section 4.5).

Overall, the gray-box reconstruction transfers to both cohorts and the observability-based lead-selection conclusions of the fixed model transport with it, even though absolute classification and MI detection require cohort-specific validation.

## 4. Discussion

### 4.1. Principal Findings

A 12-lead ECG median beat can be represented by a compact gray-box electrophysiological descriptor space that preserves both reconstruction fidelity and diagnostic information. The value of the model is not a higher classification score: a hand-crafted-feature XGBoost baseline is both interpretable and slightly more accurate (0.9200 vs. ≈0.90). What that baseline cannot provide is (i) a generative forward map that reconstructs the 12-lead signal and supports a reconstruction-quality self-check, (ii) a quantitative account, via Fisher information and observability, of how much recoverable parameter information each lead set carries and which leads to retain, and (iii) a cross-lead-consistent representation tied to lead-field geometry.

Cross-lead T-wave sign discordance, the strongest STTC discriminator, is absent from the 111 hand-crafted baseline features, so the structured surrogate captures a physiologically meaningful signal the baseline does not encode.

### 4.2. Sensor Configuration and Lead-Set Selection

For ECG sensing, the identifiability and observability analysis is the most directly useful result. A reduced lead set provably carries no more recoverable parameter information than the set it was drawn from (Section 3.7), which places a ceiling on what any reduced-lead wearable can resolve. Posing lead selection as optimal sensor selection on the Fisher information yields three-lead sets that improve parameter observability over the conventional reduced set by a wide margin under all seven noise models tested, although which three leads are optimal depends on the noise model and should not be read as a fixed recommendation. Reduced-lead ECG may nonetheless be useful for screening or wearable monitoring, provided it is interpreted as carrying a quantified, and reduced, amount of identifiable information rather than as equivalent to a standard 12-lead ECG. This is an information-optimal subset *within the standard 12-lead lead space*, not an optimization of physical electrode placement: the observability-optimal set includes precordial leads (V1/V4) that wrist- or single-patch wearables cannot readily acquire. Translating the result to a given wearable would require re-posing the selection over the electrode positions that the device can actually record; we therefore present it as guidance on *which spatial information matters*.

Our observability-based selection agrees in *form* with recent data-driven reduced-lead work. Mason et al. [18], on over 600,000 clinical ECGs, found that adding one anterior precordial lead (V3) to limb leads I and II gave the best 12-lead reconstruction ($R^{2}\approx 73\%$, single-center) and preserved MI detection (AUC 0.95). The two results converge on one point of structure—each retains lead I together with exactly one anterior precordial lead (V3 there, V4 here)—but they differ in the rest of the montage: Mason et al. hold the limb leads I and II fixed and vary only the precordial lead, so their design does not test whether lead II is needed, whereas our search over all 56 triples never selects it and the identity-noise solution keeps the septal lead V1 instead, giving one limb and two precordial leads rather than two limb and one precordial. The agreement is on the value of anterior precordial information, not on a common triple, and it is reached from a different objective: lead-field observability of the mechanistic parameters rather than waveform correlation. Presacan et al. [50] further report that single- and dual-lead reconstructions track population averages more than individual morphology, consistent with the strictly reduced information content quantified above.

#### Observability Versus Diagnostic Performance

Observability-based selection and diagnostic performance optimize different things: the selection objective asks which leads best condition the inverse problem, a diagnostic objective asks which leads best separate labels. Here the two come apart: reducing from 12 leads to lead II alone raises the Cramér–Rao bound of the ST-source almost sevenfold (3.39 to 22.76) and lowers the FIM effective rank from 4.87±0.47 to 2.76±0.17 across five seeds, but costs only about 6% of macro-AUROC (0.901 to 0.844). It follows that the AUROC column of Table 6 must not be read as a clinical ranking of montages. At three leads that column has almost no resolving power: the observability-optimal set (0.878 [0.862,0.893]), the alternate-seed optimum I+V1+V5 (0.879), the conventional clinical set II+V1+V5 (0.887 [0.871,0.902]) and the average over randomly drawn triples (0.875±0.017) all lie within one another’s intervals; the column shows only that optimizing for observability produces no detectable loss of diagnostic performance. The practical implication for sensor design is that the selection criterion must follow the intended use: a device meant to estimate mechanistic quantities should be designed on observability, where lead choice is decisive, whereas a device meant to output a four-way label is comparatively insensitive to which three leads it records and should be validated on that endpoint directly.

### 4.3. Disease-Specific Physiological Interpretation

The class–descriptor associations established in Section 3.6 are stable across seeds and consistent with standard ECG interpretation. Two points follow. First, that conduction disturbance maps to increased QRS-duration and ST/T-change to T-wave morphology is expected almost by clinical definition; we therefore treat these as *consistency checks* that the surrogate’s internal state aligns with established markers, and reserve the term “finding” for the two nontrivial, model-dependent results—the MI lead-projection vs internal-$\alpha_{\mathrm{ST}}$ dissociation and the counter-constructive STTC APD-dispersion direction. Second, the seed-instability of the internal proximal-delay descriptor against the stability of the observable QRS-duration reinforces a single theme: descriptors closer to an observable quantity are more reliable than those that depend on weakly identifiable internal parameters.

### 4.4. Systems Interpretation and Deployment

From a deployment perspective, the framework is most appropriate as a sensor-facing interpretability layer: from a 12-lead ECG it produces a reconstruction-quality measure, disease probabilities and descriptor-based explanations, and for a candidate reduced-lead configuration it quantifies which mechanistic parameters that montage can and cannot resolve, so that low-confidence reconstructions can be flagged for re-acquisition. Observability states when the encoder’s inverse is well posed: a well-conditioned Fisher information admits stable recovery; a near-singular lead set does not.

### 4.5. Limitations and Future Work

This study has several limitations. First, although we evaluated zero-shot transfer to two independent cohorts (Georgia, US; CPSC2018, China; Section 3.10) where reconstruction transferred and the lead-selection conclusions of the fixed model transported, external validation was incomplete: absolute classification degraded, ST/T-change recall was sensitive to cross-cohort labeling conventions, and **MI detection remains internally validated only**. Internally, MI also remained the most challenging class, with the largest seed-to-seed descriptor variation, and the composition of the class bears on this: PTB-XL does not annotate infarction age, and the class is defined by territory-coded infarction statements (Table S8), recognized clinically from pathological Q waves and T-wave inversion rather than from ST deviation alone. No descriptor used here measures initial QRS forces, and an eight-node source behind a rank-three lead-field represents a regional Q-wave only coarsely; a Q-wave descriptor and spatial priors for infarct localization are the natural extension. At its current MI sensitivity, I-GraphECG must not be used as a stand-alone rule-out tool for myocardial infarction: the ∼38% MI→NORM error rate means a negative output cannot safely exclude infarction. External validation was also confined to the same clean single-label criterion and relied on a SNOMED-CT-to-superclass mapping that introduces label noise. The external observability check is a transportability test of the PTB-XL decoder and lead field, not an independent validation of the selection mechanism; an independent estimate would require a surrogate trained on the external cohort. Second, the use of single median beats at 100 Hz focuses the model on beat morphology and means it does not represent rhythm, beat-to-beat variability, or arrhythmia, and the 100 Hz rate attenuates high-frequency QRS detail. Atrioventricular block, which the CD label includes, lies only partly within this scope. First-degree block is defined by the PR interval, which no descriptor measures because the P-wave is not reconstructed; the 17 fold-10 records carrying it are recalled at 0.53 against 0.59 for the class (Table S15). Second- and third-degree blocks are rhythm-level diagnoses that a median beat cannot represent; they concern 8 of the 1708 clean CD records (second-degree 5, third-degree 3), retained under their PTB-XL label. Moreover, the clean single-label design keeps the identifiability analysis free of multi-label confounding. The criterion also shapes the MI cohort: it retains 46.3% of MI records against 95.3% of NORM, so the posterior is fitted to a rarer MI than a clinical population presents (Section 3.5), and the retained MI pool carries a higher share of inferior and a lower share of anterior infarction than the excluded records under every counting convention we examined (Table S8); since anterior infarction is expressed in the precordial leads, which carry the highest relative signal-to-variance in this surrogate and enter every observability-optimal lead set under the data-derived noise models (Section 3.7), the excluded records are, if anything, the more detectable ones for this model, so the filter does not flatter the reported MI sensitivity, but neither is that sensitivity a direct estimate for unselected multi-label ECGs, which only the frozen-encoder evaluation of Section 3.9 addresses. Excluding hypertrophy keeps this first study clean but narrows the reach of the lead-field claims, since hypertrophy is where voltage and lead-field effects are most pronounced. Third, the inferred parameters are equivalent gray-box descriptors rather than true ionic, anatomical, or patient-specific tissue parameters, and the conduction graph is a fixed low-order tree rather than a patient-specific topology; the Fisher analysis is local rather than global. The lead field learned under amortized training is also not the best the parameterization admits: per-record fits with a refit lead field reach a median NRMSE about 0.1 below the trained model’s on the same records (Section 3.2), and closing that gap, for instance by fixing the gain gauge between the lead field and the nodal gains before training, is a target for future work. The specific observability-optimal montage is also noise-model-dependent (Section 3.7). Future work should extend the external validation to multi-label and MI-inclusive cohorts, move to full 10-s recordings and higher sampling rates, and test the lead-selection result, so far obtained only by simulated lead removal on 12-lead records, on prospective reduced-lead and wearable recordings.

## 5. Conclusions

I-GraphECG shows that 12-lead ECG median beats can be represented by bounded equivalent electrophysiological descriptors that reconstruct the observed signal and support interpretable disease prediction. On the PTB-XL four-class task, the model achieved a median reconstruction correlation of 0.91 and a macro-AUROC of ≈0.90 using interpretable descriptors, approaching black-box baselines while exposing disease-specific evidence that is stable across training seeds: increased QRS-duration in conduction disturbance, T-wave sign discordance in ST/T-change, and reconstruction-domain T-wave sign discordance in myocardial infarction, while the lead-projected ST descriptor is retained as an observability-limited negative example. Lead removal provably cannot add identifiable parameter information, an observability-optimal three-lead set improves parameter observability over the conventional reduced set under all seven noise models tested, and the lead-field singular values explain which parameters each lead set can resolve. I-GraphECG thus provides a sensor-facing gray-box alternative to direct waveform classification, enabling ECG disease prediction with explicit reconstruction, parameter interpretation, and observability-based lead analysis. Per-class sensitivity is uneven, and infarction is the weak class: at the neutral argmax operating point, MI recall is 0.49 and 38% of MI records are labeled NORM, so I-GraphECG must not be used as a stand-alone rule-out for myocardial infarction. Prior correction and thresholding raise that sensitivity (to 0.56 and 0.92) without moving macro-AUROC, so re-deciding does not lift the ceiling set by what the descriptors resolve about MI (Section 3.5). Under zero-shot transfer to independent US and Chinese cohorts (Section 3.10), absolute classification, tested externally with the parameter-only model, degrades, and MI could not be externally validated, so the disease-prediction component is internally demonstrated and externally unconfirmed; the reconstruction and the observability-based lead-selection conclusions of the fixed model transfer across cohorts.

## Figures and Tables

**Table 1:** PTB-XL four-class subset split and black-box baselines (fold-10 test).

Split	NORM	MI	STTC	CD	Total
train	7243	2043	1903	1353	12,542
validation	914	233	255	171	1573
test	912	256	242	184	1594
*Black-box baselines (fold-10 test): macro-AUROC*					
Hand-crafted features + XGBoost				0.9200	
ResNet1D (raw median beat)				0.9241	

**Table 2:** Reconstruction performance (fold-10 test): locked seed-42 value and mean ± SD over five seeds.

Metric	Seed-42	Mean ± SD (n=5)
scaled median NRMSE	0.401	0.383±0.010
median correlation	0.912	0.918±0.004
QRS-window NRMSE (scaled)	0.244	0.240±0.007
ST-window NRMSE (scaled)	0.490	0.486±0.028
T-window NRMSE (scaled)	0.448	0.447±0.023
P-window NRMSE (scaled)	1.628	1.304±0.239
ST mean abs. error (mV)	0.0143	0.0147±0.0011
parameter boundary rate	0.127	0.144±0.023

**Table 3:** Classification ablation using interpretable features and XGBoost (fold-10 test). Columns are the locked seed-42 macro-AUROC with its 95% patient-clustered bootstrap CI, the five-seed mean ± standard deviation (ddof =1) of macro-AUROC, and the seed-42 macro-F1. Feature groups: θ the 47 descriptors, repol. repolarization and T-wave morphology, ST lead-projected ST, aux. the auxiliary recovery descriptor.

Group	Features	Seed-42 [95% CI]	Five Seeds	F1
C0	θ (47)	0.9011 [0.887, 0.915]	0.9012±0.0031	0.706
C2	θ + repol.	0.9004 [0.886, 0.915]	0.8990±0.0029	0.698
C3	θ + ST	0.8995 [0.885, 0.914]	0.9011±0.0026	0.699
C4	θ + aux.	0.8999 [0.886, 0.915]	0.9006±0.0028	0.701
C5 (main)	θ + repol. + ST + aux. (71)	0.9014 [0.887, 0.915]	0.9001±0.0030	0.695
C6	compact heuristic (31)	0.8938 [0.879, 0.908]	0.8946±0.0031	0.676

C1, a preliminary variant of C2, is omitted, so the groups run C0, C2–C6. CIs are marginal; the paired comparison of Section 3.4 finds the five θ-based groups indistinguishable and only C6 separate.

**Table 4:** Key interpretable descriptors by class (fold-10 test; Cliff’s δ vs. NORM, mean ± SD over five seeds where a model-dependent quantity).

Class	Descriptor	Direction	Cliff’s δ
CD	QRS-duration (reconstructed)	increased	+0.53±0.02
STTC	cross-lead T-wave sign discordance (observed)	increased	+0.753
STTC	T-peak max. abs. amplitude (observed)	decreased	−0.531
MI	T-wave sign discordance (reconstruction)	increased	+0.24±0.05
MI	lead-projected ST max. abs.	unstable (neg. example)	−0.19±0.18

**Table 5:** Identifiability tiers of the 47 equivalent electrophysiological parameters (400-record subsets, five seeds; Section 2.8). Tier assignment is per parameter and uses the across-seed extremum, not the mean, so a parameter cannot enter Tier A on the strength of a single favorable seed: Tier A requires the identifiability score of Equation (13) to satisfy s≥15 in every seed and Tier C requires s<1 in every seed. The ranges overlap between tiers because the assignment uses the score, not this column. The last column is the five-seed mean of the Cramér–Rao standard deviation as a percentage of the parameter’s own admissible range, which is comparable across parameters with different units. Full per-parameter values are in Table S11. The numbers in parentheses in the Members column give how many parameters of that kind the tier contains.

Tier	Members	*n*	1σ as % of Range
A strongly identifiable	ventricular APD (3), $\tau_{\mathrm{dep}}$ (4), global gain	8	1.5–3.1
B weakly identifiable	all delays, *q* and $\tau_{\mathrm{rep}}$; remaining APD and $\tau_{\mathrm{dep}}$	33	2.4–36.3
C primarily regularized	all six $\alpha_{\mathrm{ST}}$ (ST-source)	6	150–237

**Table 6:** Lead-set identifiability, parameter recoverability, and reduced-lead disease classification. FIM effective rank (seed-42/mean ± SD over five seeds, all evaluated on the locked model’s 400-record subset) and $\alpha_{\mathrm{ST}}$ Cramér–Rao bound (CRB; scaled units, seed 42); macro-AUROC [95% patient-clustered bootstrap CI] is on the fold-10 test-set with C5 descriptors from lead-set-specific encoders (each fed only its own leads but trained to reconstruct all 12: the full-12 teacher), and the random three-lead row is the mean ± SD over ten draws (eight distinct triples); the 12-lead row uses all 12 displayed leads (Σ=I, Section 2.8). Dashes mark lead sets entering only one of the two analyses; the CIs are marginal, and the paired contrasts between the models are in Table S23.

Lead Set	FIM Eff. Rank	CRB $\alpha_{\mathrm{ST}}$	Macro-AUROC [95% CI]
12 leads	4.36/4.87±0.47	3.39	0.901 [0.887,0.915]
II+V1+V5 (clinical)	3.53/3.66±0.30	7.05	0.887 [0.871,0.902]
I+V1+V4 (observability-optimal)	3.72/4.04±0.36	6.17	0.878 [0.862,0.893]
I+V1+V5 (alternate-seed optimum)	—	—	0.879 [0.864,0.894]
I+II+V5	2.84/3.19±0.28	8.30	—
II only	2.84/2.76±0.17	22.76	0.844 [0.826,0.862]
random 3-lead	—	—	0.875±0.017

**Table 7:** External validation under zero-shot transfer (locked seed-42 model, no retraining, PTB-XL scalers reused): reconstruction and parameter-only C0 classification. Macro-AUROC and recall are over the present classes NORM/STTC/CD, MI being absent externally (see text), with recall read at the four-class argmax so that a record predicted MI counts as an error; the PTB-XL macro-AUROC is the class-matched three-class value (MI records removed, one-vs-rest AUROC of the unnormalized four-class posterior averaged over NORM/STTC/CD, exactly the external protocol; the four-class C0 value is 0.901), the PTB-XL recalls are the fold-10 C0 values under the same rule, and external 95% bootstrap CIs (1000 resamples) are in brackets.

	PTB-XL	Georgia (US)	CPSC2018 (CN)
Cleaned records	1594 (fold-10)	2586	4305
Recon median corr	0.912	0.887	0.873
Recon scaled NRMSE	0.401	0.460	0.467
macro-AUROC (3-class)	0.919	0.838 [0.821, 0.853]	0.827 [0.818, 0.837]
recall NORM/STTC/CD	0.94/0.62/0.59	0.76/0.47/0.67	0.93/0.27/0.59

## Data Availability

PTB-XL is publicly available via PhysioNet (https://physionet.org/content/ptb-xl/ (accessed on 1 September 2026)). The external-validation cohorts are also public: the Georgia database is distributed through the PhysioNet/Computing in Cardiology Challenge 2020/2021 (https://physionet.org/content/challenge-2021/ (accessed on 1 September 2026)), and CPSC2018 is available at http://2018.icbeb.org/Challenge.html (accessed on 1 September 2026). The source code, configuration files, the five trained encoder checkpoints (seeds 42 and 1–4) and the derived per-record tables that the statistical analyses consume are available under the MIT license at https://github.com/melonlink/I-GraphECG (accessed on 1 September 2026); a DOI-assigned snapshot of the same repository is archived on Zenodo at https://doi.org/10.5281/zenodo.22840835 (accessed on 1 September 2026). Every classification, identifiability, and lead-selection result reproduces from the repository alone, without raw recordings or a GPU.

## Supplementary Materials

The following supporting information can be downloaded at https://www.mdpi.com/article/10.3390/s26185933/s1.
