# Supplementary material for: I-GraphECG: Observability-Based Lead Selection and Interpretable Disease Prediction from the 12-Lead ECG Using a Gray-Box Graph Electrophysiology Surrogate
Source: Sensors (Basel). 2026 Sep 19;26(18):5933. doi: 10.3390/s26185933 (PMC13611518; doi:10.3390/s26185933)
Supplement: Supplementary file 1 [file sensors-26-05933-s001.zip › sensors-4543399-supplementary.pdf]

Supplementary Materials: I-GraphECG: Observability-Based Lead Selection and Interpretable Disease Prediction from the 12-Lead ECG Using a Gray-Box Graph Electrophysiology Surrogate

Limin Zhao *, Hongtao Xu, Pengjian Wang, Weicheng Fu and Ningning Zhang

This document provides supporting figures and tables referenced in the main text. Reproducibility details (computing environment, random seeds, official PTB-XL folds, five-seed runs, and the per-table/per-figure commands) are documented in the accompanying `REPRODUCIBILITY.md`.

1. Supplementary Figures

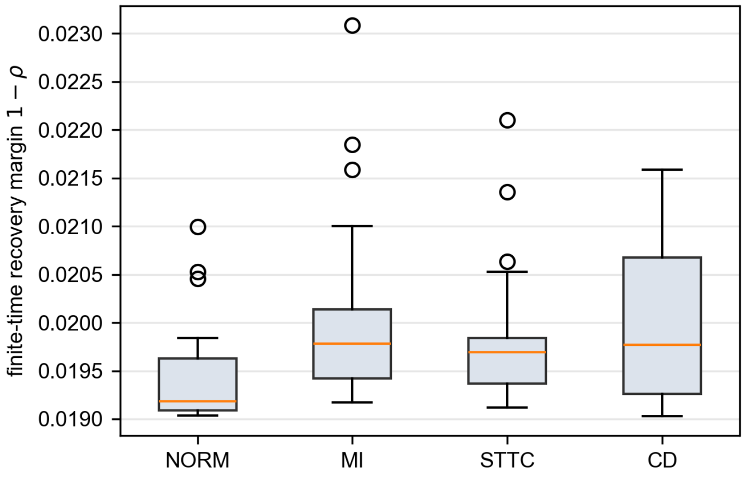

Figure S1. Auxiliary finite-time recovery descriptor. Distribution of the paced finite-time recovery margin $1 - \rho$ (one minus the recovery multiplier of the eight-node Aliev–Panfilov-style paced AP-ODE with graph diffusion) by diagnostic class. Because the paced trajectories did not converge to a periodic orbit (0% periodic convergence; see Table S1), ρ is *not* a Floquet multiplier and the descriptor supports no claim in the main text; it is reported here only as a transparency check.

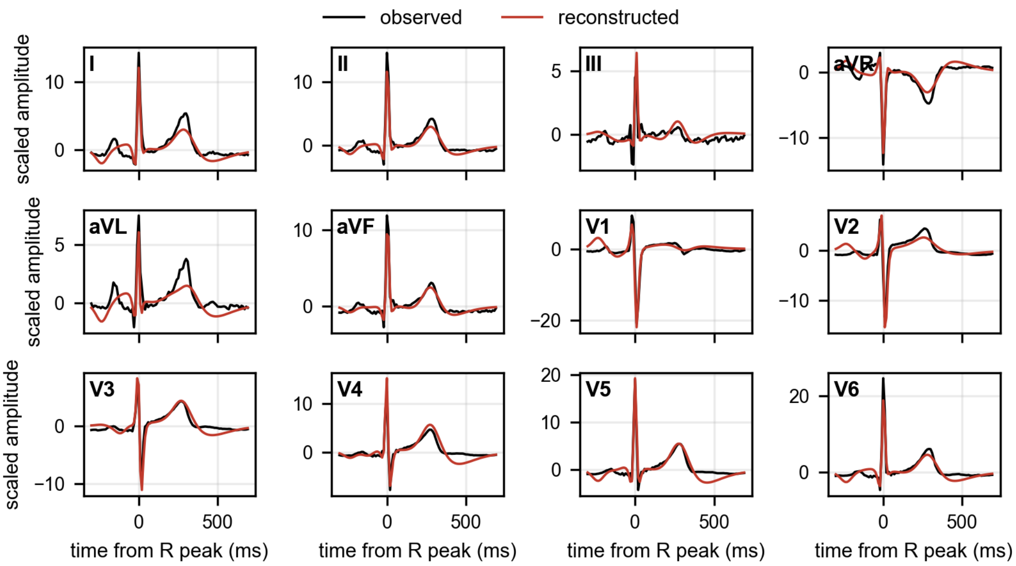

Figure S2. Full 12-lead reconstruction example. Observed (black) versus reconstructed (red) scaled median beats across all twelve standard leads for a representative NORM test record (per-record correlation 0.92). The decoder reproduces the dominant QRS and T-wave morphology across leads; the four derived limb leads are formed through the scaled-space affine Einthoven–Goldberger relations, so their traces are exact affine images of leads I and II.

2. Supplementary Tables

Table S1. Auxiliary finite-time recovery descriptor. Recovery multiplier ρ of the paced AP-ODE (mean±SD over n records per class) and the corresponding margin $1-\rho$. None of the paced trajectories converged to a periodic orbit (0% periodic convergence across all classes), so ρ is not a Floquet multiplier; the values are reported only as a transparency/negative-result check.

Class	Recovery multiplier ρ (mean±SD)	Margin $1-\rho$	n
NORM	0.9805 ± 0.0006	0.0195	20
MI	0.9799 ± 0.0010	0.0201	20
STTC	0.9802 ± 0.0008	0.0198	20
CD	0.9801 ± 0.0008	0.0199	20
All	0.9802 ± 0.0008	0.0198	80

Table S2. Single-lead (lead II) sanity-check variants. Descriptor-based four-class macro-AUROC stays near 0.84 across capacity/regularization/teacher ablations, so the single-lead performance is not merely a capacity or teacher-amortization artifact. Removing the full-12 teacher (obs mode) sharply degrades full-12 reconstruction (NRMSE 2.66), confirming the teacher's role in reconstruction rather than in disease discrimination. The internal proximal-delay descriptor is discussed in the main text (Section 3.5) as a seed-unstable negative example. The FIM effective rank is computed from each variant's own lead-II parameter estimates on the fixed 400-record subset: with the full-12 teacher it falls to 1.8–2.1, against 2.84 for the locked model (Table 6) and 2.79 without the teacher, while the θ macro-AUROC barely moves—the same dissociation between recoverable parameter information and diagnostic performance as in the lower block. The lower block retrains the three lead sets of Section 3.6 with the loss restricted to the observed leads (no teacher; the stabilized initialization of the locked recipe, head scale 0.01, gradient clip 1), evaluated with the C5 descriptors as in Table 6; the teacher rows are the Table 6 encoders. Every no-teacher macro-AUROC lies inside the patient-clustered interval of its teacher-supervised counterpart. Against teacher-supervised encoders retrained with the same initialization (0.890, 0.884, 0.857) the teacher's optimism is -0.005, $+0.007$ and $+0.011$. Macro-AUROC is from the 47 parameters alone in the upper block and from the C5 descriptors, with its 95% patient-clustered CI, in the lower block; in both blocks the FIM effective rank is computed from each row's own encoder estimates, whereas Table 6 evaluates every lead set on the locked 12-lead model's estimates.

Variant	Mode	FIM eff. rank	macro-AUROC	Observed corr.	Full-12 NRMSE
L1-baseline (full teacher)	full	2.08	0.841	0.902	0.588
L1-small encoder (full)	full	1.83	0.843	0.912	0.587
L1-regularized (full)	full	1.94	0.843	0.893	0.599
L1-no teacher (obs)	obs	2.79	0.848	0.958	2.659
Lead-set encoders of Section 3.6, with and without the full-12 teacher (C5 macro-AUROC, 95% CI)					
II+V1+V5, full teacher (Table 6)	full	3.01	0.887 [0.871, 0.902]	0.918	0.483
II+V1+V5, no teacher	obs	2.97	0.894 [0.878, 0.909]	0.938	0.572
I+V1+V4, full teacher (Table 6)	full	3.53	0.878 [0.862, 0.893]	0.926	0.452
I+V1+V4, no teacher	obs	3.08	0.877 [0.862, 0.892]	0.941	0.542
II, full teacher (Table 6)	full	1.88	0.844 [0.826, 0.862]	0.918	0.575
II, no teacher	obs	2.47	0.846 [0.827, 0.865]	0.959	2.285

Table S3. Hyperparameters, loss weights, and parameter bounds. Bounds apply to the physical parameters; the encoder outputs the unconstrained z and $\theta = \theta_{\text{center}} + \theta_{\text{radius}} \odot \tanh(z)$ maps them into $[\,\text{lo}, \text{hi}\,]$. Edge delays $d_e \geq 0$ make the activation order monotone by construction.

Group	Quantity	Value / range
Loss weights	$\lambda_{\text{rec}}/\lambda_{\text{slope}}/\lambda_{\text{QRS}}$	1.0 / 0.2 / 1.0
	$\lambda_{\text{ST}}/\lambda_{\text{T}}/\lambda_{\text{phys}}/\lambda_H$	0.5 / 0.5 / 0.05 / 0.001
$\mathcal{L}_{\text{phys}}$ internal	prior (pull-to-center)	0.1 ($\tau_{\text{dep}} \times 4$, $\tau_{\text{rep}} \times 6$)
	ST-source sparsity / boundary	1.0 / 1.0
Timing	δ_{root} (s)	[−0.24, −0.12]
	$d_{(0\to1)}$ AV (s)	[0.04, 0.20]
	$d_{(1\to2)}$ His (s)	[0.005, 0.06]
	$d_{(2\to j)}$ septum→wall (s)	[0.005, 0.07]
Repolarization	τ_{dep} (s)	[0.007, 0.026]
	τ_{rep} (s)	[0.035, 0.100]
	APD (s)	[0.08, 0.18] (atrial/AV) / [0.18, 0.45] (ventr.)
Source / gain	q	[0.05, 3.0]
	α_{ST} (a.u.)	[−0.25, 0.25]
	global gain g	[0.5, 2.0]
Decoder	derived limb leads	scaled-space affine (exact in mV)
Training	seeds	$\{42, 1, 2, 3, 4\}$ (seed-42 locked)
	encoder stabilization	head init ×0.01, grad clip 1.0
	FIM subset	400 records (100/class, fixed)
	classifier	XGBoost (primary); logistic regression (secondary)

Table S4. Per-class metrics of the main interpretable model C5 (θ + repolarization + projected ST + auxiliary descriptors) on the fold-10 test set. Upper block: the locked seed-42 model, with precision and recall from the confusion matrix (Figure 4 of the main text) and one-vs-rest AUROC and AUPRC. Lower block: mean±standard deviation across the five training seeds (42, 1, 2, 3, 4), with sensitivity and specificity at the argmax operating point.

Locked seed-42 model					
Class	AUROC	AUPRC	Precision	Recall	F_1
NORM	0.90	0.91	0.79	0.93	0.85
MI	0.88	0.67	0.69	0.49	0.57
STTC	0.93	0.75	0.72	0.61	0.66
CD	0.90	0.77	0.84	0.59	0.69
macro avg	0.90	0.78	0.76	0.65	0.70
Five seeds, mean±SD					
Class	AUROC	AUPRC	Sensitivity	Specificity	F_1
NORM	0.90 ± 0.00	0.91 ± 0.00	0.94 ± 0.01	0.66 ± 0.00	0.86 ± 0.00
MI	0.88 ± 0.01	0.67 ± 0.02	0.48 ± 0.03	0.96 ± 0.00	0.57 ± 0.03
STTC	0.93 ± 0.00	0.75 ± 0.01	0.64 ± 0.02	0.96 ± 0.00	0.68 ± 0.01
CD	0.89 ± 0.01	0.76 ± 0.01	0.58 ± 0.01	0.99 ± 0.00	0.70 ± 0.01
macro avg	0.90	0.77	0.66	0.89	0.70

3. External Validation (Supplementary)

Table S5. SNOMED-CT→four-superclass mapping used for external validation. Codes carrying hypertrophy (HYP) are excluded; codes that do not correspond to the four superclasses map to OTHER and those records are dropped (together with any multi-label record). Only the superclass-bearing codes are listed; the remaining codes present in the external cohorts (rhythm, axis, voltage, pre-excitation, etc.) map to OTHER, as does territory-coded ischemia (426434006, 425419005, 425623009), unlike the corresponding PTB-XL `ISC*` statements (Table S9). This mapping is defined by this study, may differ from the source databases' labeling conventions, and is a known noise source of cross-database transfer.

Superclass	SNOMED-CT code(s)	Term
NORM	426783006	sinus rhythm
MI	164865005	myocardial infarction
STTC	164934002; 428750005; 164930006; 429622005; 59931005; 164931005; 413444003	T-wave abnormal; nonspecific ST–T abnormality; ST interval abnormal; ST depression; T-wave inversion; ST elevation; acute myocardial ischemia
CD	270492004; 59118001; 164909002; 713426002; 713427006; 251120003; 445118002; 445211001; 698252002; 233917008; 195042002; 27885002; 6374002; 426664006	first-/second-/third-degree AV block; right/left bundle branch block (incl. incomplete/complete); fascicular blocks; nonspecific IVCD; bundle branch block
HYP	164873001; 89792004; 266249003	left/right/unspecified ventricular hypertrophy (excluded)

Table S6. External-cohort record manifests and per-class metrics under zero-shot transfer (PTB-XL-trained parameter-only C0 classifier, no retraining). AUROC/AUPRC are one-vs-rest; precision/recall/F1 at the argmax operating point. MI is absent in both external cohorts (Georgia: all MI records multi-label and excluded by single-label filtering; CPSC2018: no MI label), so MI is not evaluable externally.

Cohort	Class	n	AUROC	AUPRC	Precision/Recall	F_1
Georgia (US)	NORM	1752	0.84	0.89	0.87 / 0.76	0.81
n=2,586	STTC	542	0.80	0.56	0.58 / 0.47	0.52
	CD	292	0.87	0.73	0.71 / 0.67	0.69
	MI	0	—	—	— / —	—
CPSC2018 (CN)	NORM	918	0.85	0.53	0.40 / 0.93	0.56
n=4,305	STTC	971	0.78	0.44	0.49 / 0.27	0.35
	CD	2416	0.85	0.91	0.97 / 0.59	0.73
	MI	0	—	—	— / —	—

Disposition under the external single-label criterion (Section 3.9). Georgia: 10,344 records, of which 61% were removed for carrying a code that maps to OTHER (rhythm, axis, ectopy), 13% for hypertrophy overlap and 1% for carrying two target superclasses, leaving 2,586 (25%). CPSC2018: 6,877 records, 37% removed for an OTHER-mapped code and 1% for two target superclasses, leaving 4,305 (63%). Georgia retains 12% of its STTC-coded and 13% of its CD-coded records against all of NORM.

Table S7. Decision rules for myocardial infarction on the locked seed-42 model (C5 descriptors, fold-10 test set, $n = 1{,}594$). No rule refits the encoder. Thresholded rules select the threshold on fold 9 at the stated target sensitivity and apply it unchanged to fold 10; the thresholded rule flags MI when $P(\mathrm{MI}) \geq t$ and otherwise takes the argmax over the remaining classes. Brackets are 95% patient-clustered bootstrap intervals (1000 resamples). The prior-corrected rule rescales the posterior from the clean-subset class frequencies to the full-PTB-XL superclass frequencies and renormalizes. Macro-AUROC is essentially unchanged by every rule, so these are movements along a fixed ranking, not improvements in what the descriptors resolve.

Decision rule	MI sens. [95% CI]	MI prec.	NORM sens.	Bal. acc.	MI→NORM
argmax (main text)	0.488 [0.421, 0.559]	0.694	0.934	0.654	97
Prior-corrected argmax	0.559 [0.489, 0.624]	0.619	0.870	0.692	72
Inverse-frequency weights	0.570 [0.504, 0.635]	0.596	0.812	0.693	61
Weighted logistic regression	0.605‡	0.524	0.768	0.689	57
Threshold, target sens. 0.70	0.711 [0.646, 0.771]	0.472	0.855	0.644	58
Threshold, target sens. 0.80	0.789 [0.733, 0.841]	0.421	0.805	0.637	45
Threshold, target sens. 0.90	0.922 [0.880, 0.959]	0.295	0.629	0.556	16

‡No interval: this rule changes the classifier family rather than the operating point, so it is reported as a point estimate only. Macro-AUROC: 0.9014 (argmax), 0.9021 (prior-corrected), 0.8997 (class weights), 0.8952 (logistic regression). MI specificity—the fraction of non-MI records not flagged as MI—falls from 0.959 at argmax to 0.926 (class weights), 0.848, 0.792 and 0.579 at the three thresholds. At the 0.90 target, 799 of 1594 records are flagged as MI.

Table S8. Myocardial-infarction subclass composition of the records the inclusion criterion retains versus those it excludes, under four counting conventions. PTB-XL annotates each record with one or more MI subclass codes (AMI anterior, IMI inferior, LMI lateral, PMI posterior), each carrying a likelihood in $[0, 100]$; shares are percentages of subclass *tags*, not of records, and n is the number of tags counted. "Dedup" counts each subclass at most once per record; "all codes" counts every occurrence. The relative comparison is stable—under every convention the retained pool carries a higher IMI and a lower AMI share than the excluded pool—but the absolute composition is not: the retained pool is inferior-dominant under the first three conventions and anterior-dominant under the fourth. The main text therefore states only the relative comparison.

	tags counted		AMI share (%)		IMI share (%)	
Counting convention	retained	excluded	retained	excluded	retained	excluded
Dedup, all codes	3078	3489	40.3	52.7	56.6	43.8
Every code, all codes	3224	3639	43.0	54.7	54.0	42.0
Dedup, likelihood ≥ 50	2238	2698	46.5	56.3	50.8	41.0
Dedup, likelihood $= 100$	1580	1887	51.4	60.9	47.0	37.2

LMI and PMI together account for 1.7–3.5% of tags in every cell and are omitted for space. Retained = the 2,532 MI records of the clean subset; excluded = MI-coded records removed by the multi-label or hypertrophy criteria.

Table S9. What the ST/T-change (STTC) label contains in each cohort, as a percentage of that cohort's clean single-label STTC records. PTB-XL is annotated with SCP-ECG statements ($n = 2400$); the external cohorts are annotated with SNOMED-CT codes and are shown as mapped by the study map ($n = 542$ Georgia, $n = 971$ CPSC2018). Records may carry more than one code, so columns need not sum to 100. The three label sets are not the same object. PTB-XL is dominated by T-wave abnormality and Georgia carries substantial T-wave content alongside a nonspecific ST-T majority, whereas CPSC2018 consists of ST depression and ST elevation and contains no T-wave-coded record at all. This is the ordering the external STTC recalls follow (0.47 Georgia, 0.27 CPSC2018), and it is consistent with cross-lead T-wave sign discordance being the surrogate's strongest STTC discriminator.

Label family	PTB-XL (%)	Georgia (%)	CPSC2018 (%)
T-wave abnormality or inversion	55.1	49.3	0.0
Non-specific ST or ST-T change	19.8	64.8	0.0
Territory-coded ischemia	22.0	0.0	0.0
ST depression	n/a†	0.9	81.0
ST elevation	n/a†	2.6	19.3
Records	2400	542	971

†PTB-XL annotates non-specific ST depression (`STD_`) and ST elevation (`STE_`) as *form* statements carrying no `diagnostic_class`, so neither belongs to the STTC diagnostic superclass under the standard aggregation. The mismatch with CPSC2018 is therefore structural rather than a matter of degree: its STTC pool has essentially no counterpart in the superclass the model was trained on. PTB-XL families: T-wave = `NDT`; non-specific ST = `NST_`; territory-coded ischemia = `ISCA*`, `ISCI*`, `ISCLA`, `ISC_`. The Georgia 0.0 for territory-coded ischemia is a property of the study map, not of the cohort's annotation: Georgia codes territory ischemia as anterior (426434006), inferior (425419005) and lateral (425623009), and the map sends all three to OTHER, so every record carrying one of them is dropped by the exactly-one-superclass criterion, whereas PTB-XL's `ISC*` statements belong to STTC. The map was fixed before evaluation and is not revised here. Neither cohort annotates infarction territory or age.

Table S10. Einthoven–Goldberger coefficients in the lead-wise robust-scaled space in which the decoder operates, computed from the locked training scalers by Equation (9) of the main text, together with the physical coefficients they replace. The last three columns give the absolute discrepancy that would result from applying the physical coefficients to scaled signals—the implementation error this convention is stated to prevent—evaluated on the observed fold-10 records ($n = 1{,}594$, 159,400 samples per lead). The median discrepancy is small in every derived lead, but the tail is not, so the choice is consequential for individual beats rather than for aggregate statistics.

	scaled-space $(\tilde{a}, \tilde{b}, \tilde{c})$			physical (a, b)		|discrepancy| (mV)		
Lead	I	II	const	I	II	median	p99.9	max
III	−1.076	+1.422	−0.024	−1	+1	0.0086	0.297	0.593
aVR	−0.451	−0.596	−0.021	$-\frac{1}{2}$	$-\frac{1}{2}$	0.0021	0.070	0.159
aVL	+1.394	−0.921	−0.039	+1	$-\frac{1}{2}$	0.0061	0.252	0.440
aVF	−0.509	+1.346	−0.040	$-\frac{1}{2}$	+1	0.0088	0.284	0.531

The scaled-space coefficients reproduce the analytic values of Equation (9) to 4.4×10^{-5}, and reduce to the physical coefficients exactly when the scaler is the identity (unit interquartile range, zero median). Relative RMS error against the observed derived leads on fold 10 is unchanged by working in scaled coordinates (III 0.1131 scaled versus 0.1131 physical; aVL 0.0789 versus 0.0796; aVF 0.0776 versus 0.0780; aVR 0.0397 versus 0.0407) but rises to 0.342, 0.280, 0.343 and 0.076 if the physical coefficients are applied in scaled coordinates. Because the waveform loss terms are computed over all twelve displayed leads, the derived leads enter the training objective, so any statement that the Fisher information is unaffected by the convention applies to a fixed trained decoder, not across training runs.

Table S11. All 47 equivalent electrophysiological parameters: admissible bounds, units, five-seed identifiability (mean and across-seed minimum), the Cramér–Rao standard deviation as a percentage of the parameter's own admissible range, the identifiability tier, the parameter's strongest Fisher-coupling partner at seed 42 (with the number of seeds, out of five, in which that partner is the strongest), and how the manuscript uses the parameter. No individual parameter is interpreted directly: interpretation is attached to derived descriptors (Section 3.5), and the use column records the route: D, enters a stated θ-domain descriptor; I, enters the reconstruction or a computed but uninterpreted descriptor only; N, negative example only; L, observability-limited and reported as not a marker; –, not used. Identifiability scores and Cramér–Rao bounds are computed on radius-scaled parameter columns, so they are dimensionless and comparable across parameters; the percentage column is the interpretable summary. Tier A requires $s \geq 15$ in every seed, Tier C $s < 1$ in every seed. Each seed's scores are computed on the 400-record subset drawn with that run's seed; recomputing all five on the locked model's subset leaves the Tier A and Tier C memberships identical and moves the percentage ranges by at most two points. A group-averaged criterion (tiering by the mean and coefficient of variation of each parameter group) was rejected: averaging over the eight members of a group cancels their spread—per-parameter coefficients of variation reach 83%—and would admit 25 parameters to Tier A, including parameters whose one-standard-deviation uncertainty is a third of their admissible range. Ordered by tier, then by index.

#	parameter	group	lo	hi	unit	$\bar{s}$	min s	1σ %	tier	partner ($n/5$)	use
11	APD^3	APD	0.18	0.45	s	20.71	18.50	2.4	A	α_{ST}^1 (2)	D
13	APD^5	APD	0.18	0.45	s	23.29	15.67	2.4	A	α_{ST}^3 (2)	D
15	APD^7	APD	0.18	0.45	s	28.10	19.91	1.9	A	α_{ST}^5 (3)	D
19	τ_{dep}^3	tau_dep	0.007	0.026	s	30.45	24.18	1.7	A	q3 (1)	I
20	τ_{dep}^4	tau_dep	0.007	0.026	s	30.79	21.41	1.7	A	τ_{dep}^5 (2)	I
21	τ_{dep}^5	tau_dep	0.007	0.026	s	34.39	27.51	1.5	A	τ_{dep}^4 (3)	I
23	τ_{dep}^7	tau_dep	0.007	0.026	s	33.65	22.66	1.6	A	τ_{dep}^4 (2)	I
46	`global_gain`	global_gain	0.5	2	1	15.92	15.60	3.1	A	q5 (1)	I
0	`delta_root`	delta_root	−0.24	−0.12	s	9.37	3.43	6.8	B	`edge_prox0` (5)	–
1	`edge_prox0`	edge_prox	0.04	0.2	s	9.27	4.55	6.3	B	`delta_root` (4)	N
2	`edge_prox1`	edge_prox	0.005	0.06	s	8.93	2.32	10.2	B	`edge_leaf2` (1)	N
3	`edge_leaf0`	edge_leaf	0.005	0.07	s	27.79	3.35	4.2	B	`edge_leaf2` (1)	I
4	`edge_leaf1`	edge_leaf	0.005	0.07	s	30.32	3.37	4.2	B	`edge_leaf2` (1)	I
5	`edge_leaf2`	edge_leaf	0.005	0.07	s	30.75	3.38	4.2	B	`edge_leaf4` (2)	I
6	`edge_leaf3`	edge_leaf	0.005	0.07	s	23.38	3.37	4.5	B	`edge_leaf1` (1)	I
7	`edge_leaf4`	edge_leaf	0.005	0.07	s	27.46	3.37	4.3	B	`edge_leaf2` (1)	I
8	APD^0	APD	0.08	0.18	s	1.55	1.13	36.3	B	q0 (2)	–
9	APD^1	APD	0.08	0.18	s	2.89	1.93	18.2	B	`edge_prox0` (1)	–
10	APD^2	APD	0.18	0.45	s	10.76	2.95	7.2	B	α_{ST}^0 (3)	D
12	APD^4	APD	0.18	0.45	s	29.67	7.57	2.6	B	α_{ST}^2 (2)	D
14	APD^6	APD	0.18	0.45	s	10.64	3.05	6.6	B	APD^3 (1)	D
16	τ_{dep}^0	tau_dep	0.007	0.026	s	2.05	0.94	28.2	B	q0 (4)	I
17	τ_{dep}^1	tau_dep	0.007	0.026	s	5.81	2.28	14.4	B	q1 (4)	I
18	τ_{dep}^2	tau_dep	0.007	0.026	s	22.05	1.68	7.9	B	q2 (4)	I
22	τ_{dep}^6	tau_dep	0.007	0.026	s	27.10	10.15	2.4	B	q6 (2)	I
24	τ_{rep}^0	tau_rep	0.035	0.1	s	1.93	1.29	29.4	B	q0 (4)	I
25	τ_{rep}^1	tau_rep	0.035	0.1	s	4.04	2.38	13.6	B	q1 (3)	I
26	τ_{rep}^2	tau_rep	0.035	0.1	s	5.09	1.39	15.0	B	q2 (2)	I
27	τ_{rep}^3	tau_rep	0.035	0.1	s	10.07	9.30	5.0	B	q3 (1)	I
28	τ_{rep}^4	tau_rep	0.035	0.1	s	13.40	3.50	5.6	B	q4 (2)	I
29	τ_{rep}^5	tau_rep	0.035	0.1	s	10.97	7.59	5.0	B	τ_{rep}^4 (4)	I
30	τ_{rep}^6	tau_rep	0.035	0.1	s	5.24	1.32	14.3	B	τ_{rep}^3 (1)	I
31	τ_{rep}^7	tau_rep	0.035	0.1	s	13.34	9.85	3.9	B	τ_{rep}^4 (1)	I
32	q0	q	0.05	3	1	13.15	7.78	4.3	B	τ_{dep}^0 (5)	I
33	q1	q	0.05	3	1	25.01	14.25	2.5	B	τ_{dep}^1 (3)	I
34	q2	q	0.05	3	1	27.54	10.13	2.9	B	τ_{dep}^2 (1)	I
35	q3	q	0.05	3	1	16.65	11.06	3.3	B	q5 (1)	I
36	q4	q	0.05	3	1	15.33	10.32	3.6	B	q7 (1)	I
37	q5	q	0.05	3	1	15.88	11.47	3.3	B	q7 (2)	I
38	q6	q	0.05	3	1	18.96	13.74	2.8	B	q3 (2)	I
39	q7	q	0.05	3	1	17.91	11.04	3.2	B	q4 (1)	I
40	α_{ST}^0	alpha_ST	−0.25	0.25	a.u.	0.39	0.18	162.4	C	α_{ST}^5 (2)	L
41	α_{ST}^1	alpha_ST	−0.25	0.25	a.u.	0.24	0.14	236.5	C	α_{ST}^3 (1)	L
42	α_{ST}^2	alpha_ST	−0.25	0.25	a.u.	0.29	0.16	221.2	C	α_{ST}^5 (1)	L
43	α_{ST}^3	alpha_ST	−0.25	0.25	a.u.	0.22	0.18	233.8	C	α_{ST}^2 (2)	L
44	α_{ST}^4	alpha_ST	−0.25	0.25	a.u.	0.35	0.23	150.5	C	α_{ST}^1 (1)	L
45	α_{ST}^5	alpha_ST	−0.25	0.25	a.u.	0.26	0.15	212.1	C	α_{ST}^2 (1)	L

$\bar{s}$ and min s are the five-seed mean and minimum identifiability score. Every α_{ST} component sits in Tier C, where the posterior standard deviation exceeds the admissible range, so those values are set by the prior rather than by the data.

Table S12. Fidelity of the reconstruction-domain descriptors against their observation-domain twins (Pearson r, five-seed mean±SD, fold-10 test set, $n = 1{,}594$). The eight best- and eight worst-recovered of the 37 pairs are shown. The split is by descriptor kind rather than by magnitude: 21 of the 24 amplitude, area and slope descriptors reach $r \geq 0.80$, while all 12 timing and shape descriptors and the single polarity descriptor (reconstruction-domain T-wave sign discordance, $r = 0.37 \pm 0.12$) fall below 0.50.

best recovered		worst recovered	
descriptor	r	descriptor	r
`STslope_std`	0.952 ± 0.007	`Tptime_std`	0.302 ± 0.039
`Tabsarea_mean`	0.951 ± 0.008	`Tasym_mean`	0.271 ± 0.052
`STslope_range`	0.934 ± 0.010	`Twidth_maxabs`	0.267 ± 0.028
`STmean_mean`	0.930 ± 0.017	`Twidth_range`	0.267 ± 0.023
`STmean_std`	0.928 ± 0.008	`Tasym_range`	0.230 ± 0.030
`Tarea_std`	0.922 ± 0.009	`Tptime_range`	0.189 ± 0.038
`STslope_mean`	0.921 ± 0.005	`Twidth_mean`	0.115 ± 0.069
`Tarea_mean`	0.915 ± 0.011	`Tptime_maxabs`	0.070 ± 0.034

The amplitude-derived `Tlowamp` family spans both bands and is the one exception to the kind-based split; `Tlowamp_maxabs` is set by the flattest lead rather than the largest, which is why it behaves unlike its siblings. Pearson and Spearman disagree for the `STslope` family, whose high linear correlation is partly tail-driven. Stratification by reconstruction quality (Section 3.2): pooled over classes, accuracy declines across NRMSE quartiles (0.820 $\rightarrow$ 0.715) because NORM concentrates in the well-reconstructed quartiles (fraction NORM 0.635 $\rightarrow$ 0.382); the per-record Spearman correlation between scaled NRMSE and the predicted probability of the true class is $+0.231 \pm 0.034$ for MI ($p < 0.05$ in 5/5 seeds) and -0.146 ± 0.037 for NORM. Within MI, recall rises from 0.375 in the best-reconstructed quartile to 0.634 in the worst ($\Delta = +0.259 \pm 0.065$, 5/5 seeds), and 67% of the MI$\rightarrow$NORM errors fall in the two best-reconstructed MI quartiles.

Table S13. Multi-label PTB-XL evaluation with the surrogate frozen. The locked seed-42 encoder and decoder are applied zero-shot to the canonical superdiagnostic task (21,388 records with at least one diagnostic superclass, official fold split, five labels including the hypertrophy class the main analysis excludes); the frozen PTB-XL training scaler is reused and nothing about the surrogate is refitted. Only the downstream one-vs-rest classifier is fitted, on the multi-label training folds. Brackets are 95% patient-clustered bootstrap intervals over the 1877 fold-10 patients.

	clean single-label ($n = 1{,}594$)	all ≥ 1 superclass ($n = 2{,}158$)	> 1 superclass ($n = 508$)
Recon. median NRMSE (scaled)	0.4009	0.4068	0.4440
Recon. median correlation	0.9115	0.9069	0.8788
Five-label macro-AUROC, encoder frozen, fold 10 ($n = 2158$)			
θ only (C0)			0.8941 [0.8850, 0.9023]
Full descriptor set (C5)			0.8929 [0.8834, 0.9015]
Per-label AUROC, θ only			
NORM			0.9161 [0.9031, 0.9274]
MI			0.8700 [0.8507, 0.8875]
STTC			0.9174 [0.9031, 0.9307]
CD			0.8784 [0.8564, 0.8977]
HYP			0.8888 [0.8681, 0.9075]

Degradation on multi-label records relative to the 1,650 single-superclass fold-10 records (median NRMSE 0.4001; they include the hypertrophy-only records that the clean column excludes): $+0.044$ [$+0.028$, $+0.061$] NRMSE and -0.033 [-0.042, -0.025] correlation, grading with label cardinality (0.4001, 0.4399, 0.4462 for one, two, and three or more superclasses). Hypertrophy is discriminated at 0.889 although the surrogate was never trained on it.

Table S14. Per-record oracle fits on the fold-10 test set ($n = 1594$, locked seed-42 lineage), the like-for-like comparison behind Section 3.1. Each row optimizes the 47 parameters record by record under the training objective (Adam, early stopping on the in-sample median NRMSE); "lead field refit" also optimizes the shared lead field $H = AB$. The lower block refits the lead field on one interleaved half of the fold and applies it, frozen, to the other half. Scaled coordinates; window NRMSE as in Table 2.

Fit	median NRMSE	median corr.	QRS	ST	T	epochs
Encoder (amortized, locked model)	0.401	0.912	0.244	0.490	0.448	—
Per-record, lead field locked, encoder init.	0.393	0.917	0.224	0.411	0.415	200
Per-record, lead field locked, center init.	0.390	0.921	0.217	0.405	0.412	1175
Per-record, lead field refit	0.295	0.949	0.203	0.345	0.306	9075
Lead-field transfer between the two halves of the fold ($n = 797$ each)						
Refit on half A (in-sample)	0.302	0.946				6750
Half-A lead field frozen, fit on half B	0.312	0.942				3600
Locked lead field, fit on half B	0.393	0.920				1250

The encoder lies within 0.011 NRMSE of the per-record optimum of its own decoder (oracle better on 73–78% of records; the gap is 0.005 for NORM and 0.016–0.026 for the abnormal classes). The refit lead field, whose leading singular values are about three times those of the locked one, transfers between halves, so the remaining gap is attributable to the lead field learned under amortized training rather than to the parameterization or the encoder.

Table S15. Atrial-window control. The locked seed-42 recipe retrained with one additional loss term over the atrial window (-300 to -100 ms) at two weights (the ST/T weight and four times it), evaluated with the same pipeline as the locked model on the fold-10 test set. d_0 is the sinoatrial-to-atrioventricular conduction delay, q_0 the atrial source gain, s the identifiability score of Equation (13) of the main text.

	locked	atrial term, 0.5	atrial term, 2.0
P-window NRMSE	1.628	0.956	0.882
QRS / ST / T window NRMSE	0.244 / 0.490 / 0.448	0.249 / 0.502 / 0.484	0.242 / 0.445 / 0.498
Overall median NRMSE / corr.	0.401 / 0.912	0.381 / 0.919	0.369 / 0.922
C5 macro-AUROC	0.9014 [0.887, 0.915]	0.8984 [0.884, 0.913]	0.9034 [0.890, 0.916]
CD recall / first-degree AV-block recall (n=17)	0.587 / 0.529	0.576 / 0.529	0.609 / 0.529
Across-record SD of d_0 (s)	0.0040	0.0042	0.0039
Records with q_0 at its lower bound	0.4%	19%	89%
s of δ_{root} / d_0	13.7 / 10.0	3.7 / 4.2	2.0 / 2.5

The extra term lowers the atrial-window error by removing the locked model's spurious deflection there, not by fitting the P wave: the atrial source gain collapses to its bound, the atrioventricular delay remains constant across records, and the identifiability of the atrial timing parameters falls. Atrial timing is therefore not recoverable from this surrogate.

Table S16. Paired patient-clustered bootstrap over all fifteen contrasts between the feature groups of Table 3 (fold-10 macro-AUROC, XGBoost, locked seed-42 model, 2000 replicates). Δ is the macro-AUROC of the first group minus the second; p values are two-sided, with the Benjamini–Hochberg correction of Section 2.11. Among the five θ-based groups no contrast is significant (smallest uncorrected $p = 0.108$); C6 separates from C5, C0 and C2 after correction, and the C5–C6 contrast also survives a Bonferroni threshold.

Contrast	Δ	95% CI	p	p_{BH}	significant (BH, 0.05)
C5 vs C6	$+0.0076$	$[+0.0026, +0.0128]$	0.002	0.030	yes
C0 vs C6	$+0.0073$	$[+0.0020, +0.0129]$	0.009	0.050	yes
C2 vs C6	$+0.0067$	$[+0.0017, +0.0117]$	0.010	0.050	yes
C4 vs C6	$+0.0061$	$[+0.0009, +0.0116]$	0.021	0.079	no
C3 vs C6	$+0.0058$	$[+0.0003, +0.0115]$	0.038	0.114	no
C0 vs C3	$+0.0016$	$[-0.0003, +0.0034]$	0.108	0.270	no
C3 vs C5	-0.0019	$[-0.0046, +0.0008]$	0.185	0.396	no
C0 vs C4	$+0.0012$	$[-0.0009, +0.0032]$	0.246	0.435	no
C4 vs C5	-0.0015	$[-0.0041, +0.0011]$	0.261	0.435	no
C2 vs C5	-0.0010	$[-0.0032, +0.0012]$	0.395	0.593	no
C2 vs C3	$+0.0009$	$[-0.0021, +0.0037]$	0.546	0.745	no
C0 vs C2	$+0.0007$	$[-0.0020, +0.0034]$	0.615	0.769	no
C2 vs C4	$+0.0005$	$[-0.0020, +0.0031]$	0.682	0.787	no
C3 vs C4	-0.0004	$[-0.0024, +0.0017]$	0.759	0.813	no
C0 vs C5	-0.0003	$[-0.0030, +0.0025]$	0.875	0.875	no

Table S17. Per-lead pre-QRS variability of the median beats (training folds; DC-removed standard deviation over the first 20 samples, −300 to −110 ms, a segment that contains baseline noise and the atrial deflection the surrogate does not model). In millivolts it is comparable across leads and lowest in aVL; relative to each lead's own robust scale, which is what the Fisher information weights, V2–V5 carry a 1.5–2.1× smaller unmodeled pre-QRS standard deviation than the limb leads and V1 because their signal amplitude is larger. The empirical Σ of Section 2.8 is diagonal, with the squares of the scaled column as its entries.

Lead	pre-QRS SD (mV)	robust IQR (mV)	pre-QRS SD (scaled)	precordial
I	0.0274	0.0511	0.537	no
II	0.0390	0.0675	0.578	no
III	0.0250	0.0475	0.526	no
aVR	0.0314	0.0567	0.554	no
aVL	0.0176	0.0367	0.481	no
aVF	0.0295	0.0502	0.589	no
V1	0.0251	0.0459	0.548	yes
V2	0.0294	0.1035	0.284	yes
V3	0.0313	0.1096	0.286	yes
V4	0.0269	0.0914	0.294	yes
V5	0.0226	0.0694	0.326	yes
V6	0.0210	0.0539	0.389	yes

Table S18. Design-fold check of the three-lead selection. The exhaustive identity-noise selection of Section 2.9 (eight independent leads, 56 triples, $F_\lambda = F + \lambda I$) repeated for every seed with the class-balanced 400-record design subset drawn from the test fold 10 (the published protocol), from the validation fold 9 and from the training folds 1–8. The D- and A-optimal sets do not depend on the design fold at any seed; the D-optimal margin over the clinical set II+V1+V5 (log det units) moves by at most 0.21 and the clinical set's rank among the 56 triples by at most two places; the E-optimal set, which depends on the smallest, ridge-dominated eigenvalue, changes with the design subset at seeds 42 and 3.

Seed	Design subset	D-optimal	A-optimal	E-optimal	Margin over II+V1+V5	Clinical rank /56
42	fold 10 (test)	I+V1+V4	I+V1+V4	V1+V2+V5	9.98	22
42	fold 9 (validation)	I+V1+V4	I+V1+V4	I+V1+V4	10.01	22
42	folds 1-8 (training)	I+V1+V4	I+V1+V4	V1+V2+V5	9.85	22
1	fold 10 (test)	I+V1+V5	I+V1+V4	V1+V2+V4	8.05	5
1	fold 9 (validation)	I+V1+V5	I+V1+V4	V1+V2+V4	7.85	5
1	folds 1-8 (training)	I+V1+V5	I+V1+V4	V1+V2+V4	7.87	5
2	fold 10 (test)	I+V1+V5	I+V1+V4	V1+V2+V4	6.97	9
2	fold 9 (validation)	I+V1+V5	I+V1+V4	V1+V2+V4	6.80	8
2	folds 1-8 (training)	I+V1+V5	I+V1+V4	V1+V2+V4	6.82	8
3	fold 10 (test)	I+V1+V4	I+V1+V5	V1+V2+V4	10.78	13
3	fold 9 (validation)	I+V1+V4	I+V1+V5	V1+V2+V5	10.62	15
3	folds 1-8 (training)	I+V1+V4	I+V1+V5	V1+V2+V5	10.66	14
4	fold 10 (test)	I+V1+V5	I+V1+V5	V1+V2+V6	8.67	8
4	fold 9 (validation)	I+V1+V5	I+V1+V5	V1+V2+V6	8.58	8
4	folds 1-8 (training)	I+V1+V5	I+V1+V5	V1+V2+V6	8.67	8

Table S19. Transfer of the observability analysis of the locked model across cohorts (locked seed-42 model, no retraining, PTB-XL scalers reused; 400-record class-balanced subsets, Section 2.8). FIM spectral-entropy effective rank per lead set and the greedy D-optimal three-lead set among the eight independent leads. Effective rank measures eigenvalue spread, not a dimension count, and is not strictly monotone in lead number (Section 3.6); the 12-lead value uses all twelve displayed leads under $\Sigma = I$. The lead-field singular spectrum is a property of the fixed decoder and is identical across cohorts by construction.

	PTB-XL	Georgia (US)	CPSC2018 (CN)
Eff. rank, 12 leads	4.36	4.64	3.91
Eff. rank, I+V1+V4 (obs.-optimal)	3.72	4.08	3.56
Eff. rank, II+V1+V5 (clinical)	3.53	3.65	3.17
Eff. rank, II only	2.84	2.70	2.29
D-optimal three-lead set	I+V1+V4	I+V1+V4	I+V1+V4

Table S20. Structural ablation of the surrogate (seed 42, one variant at a time, every other setting the locked recipe). Fold-10 reconstruction (scaled median NRMSE, median correlation, QRS-window NRMSE, parameter boundary rate), macro-AUROC of the parameter-only (C0) and main (C5) classifiers with 95% patient-clustered bootstrap CI for C5 (the C0 intervals are of the same width), the FIM effective rank on the design subset at 12 leads and for lead II alone, and two consistency measures that the locked design fixes by construction: the relative error of the reconstructed limb leads III, aVR, aVL and aVF against the Einthoven–Goldberger relations in physical units (median over records, mean over the four leads), and the fraction of test records in which some node activates before its parent on the conduction tree. The rank variants scale the random initial lead field so that its entry variance equals the rank-3 recipe's ($s = 0.3\,(3/r)^{1/4}$); with the unscaled initialization the rank-5 encoder stalls in the tanh-saturation regime the recipe was tuned against. The rank-8 encoder, whose frozen random lead field gives no validation gain during the 20 warm-up epochs, needs the early-stopping patience extended by that warm-up (40 instead of 20 epochs) or it stops at the first trainable epoch, and it leaves the saturation regime only after about 90 epochs, so it was given 400 instead of 150 epochs. The free-activation-time variant keeps 47 parameters (eight node activation times replace the root onset and seven edge delays); its descriptors that read edge delays are then read on absolute times, so its C5 value is not like-for-like.

Variant	NRMSE	corr.	QRS	bound.	C0 AUROC	C5 AUROC [95% CI]	eff. rank 12 / II	limb err.	order viol.
locked model	0.401	0.912	0.244	0.127	0.901	0.901 [0.887, 0.915]	4.36 / 2.84	0.000	0.000
lead-field rank 1	0.707	0.622	0.636	0.142	0.855	0.856 [0.838, 0.871]	2.87 / 2.87	0.000	0.000
lead-field rank 5	0.341	0.931	0.218	0.109	0.897	0.897 [0.882, 0.912]	5.98 / 3.23	0.000	0.000
lead-field rank 8 (unconstrained)	0.371	0.917	0.194	0.058	0.903	0.904 [0.891, 0.917]	6.50 / 3.17	0.000	0.000
no physics loss	0.397	0.911	0.249	0.304	0.900	0.897 [0.882, 0.912]	4.42 / 2.82	0.000	0.000
Einthoven coeff. in scaled space	0.405	0.907	0.262	0.083	0.901	0.899 [0.884, 0.913]	4.46 / 2.93	0.341	0.000
no exact limb relations (12-row lead field)	0.344	0.928	0.237	0.128	0.903	0.901 [0.886, 0.914]	5.94 / 3.48	0.022	0.000
free activation times (no graph)	0.367	0.923	0.241	0.094	0.893	0.894 [0.878, 0.908]	4.40 / 1.83	0.000	0.982

Table S21. Do the classifiers depend on weakly identifiable directions? Upper block: every fold-10 parameter estimate of the locked model is displaced along a random combination of the m least-informative eigendirections of the population Fisher information (radius-scaled coordinates, Gaussian coefficients of the stated standard deviation in radius units, clipped to the admissible box, five draws per record), or, as a control, along the six most-informative directions; the columns give the median parameter displacement, the change of the reconstruction it causes, and the effect on the unchanged C0 and C5 classifiers (argmax flip rate and change of macro-AUROC; baseline C0 0.9011, C5 0.9014). Lower block: an alternative inverse, the per-record refit of every fold-10 record with the decoder fixed (the center-initialized, lead-field-locked fit of Table S14); its features are scored by the encoder-trained classifiers, and the last line gives the record-level agreement of each parameter between the two inverses, grouped by the tiers of Table S11.

Directions	step	displacement	$\lvert\Delta$NRMSE$\rvert$	C0 flips	C0 ΔAUROC	C5 flips	C5 ΔAUROC
least inf. 6	0.25	0.47	0.0007	0.155	-0.0252	0.133	-0.0192
least inf. 6	0.50	0.89	0.0019	0.243	-0.0541	0.221	-0.0453
least inf. 12	0.25	0.71	0.0014	0.265	-0.0508	0.244	-0.0448
least inf. 12	0.50	1.32	0.0069	0.383	-0.0868	0.373	-0.0775
least inf. 24	0.25	1.05	0.0190	0.418	-0.0910	0.382	-0.0821
least inf. 24	0.50	1.97	0.1271	0.626	-0.1540	0.605	-0.1411
most inf. 6	0.25	0.55	0.9923	0.340	-0.0818	0.348	-0.0818
most inf. 6	0.50	1.05	1.6004	0.499	-0.1179	0.509	-0.1223

Alternative inverse (per-record refit, decoder fixed): median NRMSE 0.390 (encoder 0.401)
C0 on refit features 0.7110, argmax agreement with the encoder 0.349; C5 0.7155, agreement 0.269
per-parameter agreement of the two inverses (median Pearson r over records, by tier):
Tier A: 0.72; Tier B: 0.35; Tier C: 0.41

Table S22. Three-lead D-optimal selection under seven noise models (exhaustive search over the 56 subsets of the eight independent leads; same five checkpoints and 400-record design subset as Section 3.6; $\lambda = 10^{-3}$). Upper block: the optimum at seed 42 and the optima at seeds 1–4 (with their counts), the seed-42 margin of $\log\det F_\lambda$ over the clinical set II+V1+V5 and the clinical set's rank, whether lead II enters any seed's optimum, and whether every optimum contains one of V2–V5. The empirical full covariance is the pre-QRS lead covariance of the training folds (largest off-diagonal lead correlation 0.88); the artifact models scale the noise variance of one lead group by four; the AR(1) model whitens each lead in time. The empirical diagonal model is normalized over the eight-lead pool here, so its seed-42 margin (32.34) differs in the second decimal from the twelve-lead normalization of Section 3.6 (32.38). Lower block (seed 42): the best set within three wearable-feasible families, with its loss of log det against the unconstrained optimum and its margin over the clinical set in parentheses.

Noise model	optimum (42)	optima, seeds 1–4 (count)	margin	clin. rank	II ever	V2–V5 always
identity (Sect. 3.6)	I+V1+V4	I+V1+V5 (3), I+V1+V4	+9.98	22	no	yes
empirical diagonal (Sect. 3.6)	V2+V3+V5	V2+V4+V5 (2), I+V2+V4, V1+V2+V4	+32.34	48	no	yes
empirical full covariance	I+V2+V4	I+V2+V4 (4)	+22.58	38	no	yes
limb-lead variance ×4	V1+V2+V5	V1+V2+V5 (4)	+17.59	21	no	yes
precordial variance ×4	I+II+V1	I+II+V1 (4)	+23.41	22	yes	no
AR(1) in time, $\rho = 0.8$	V1+V2+V5	I+V1+V5 (3), I+V1+V4	+9.55	20	no	yes
empirical full $\otimes$ AR(1)	I+V2+V4	II+V2+V4, I+V2+V4 (3)	+21.11	39	yes	yes

Wearable-feasible families, seed 42: best set (loss vs. optimum; margin over clinical)

Family	identity	empirical diagonal	empirical full $\otimes$ AR(1)
at most one precordial lead	I+II+V1 (-9.69; +0.29)	I+II+V2 (-23.40; +8.94)	I+II+V2 (-18.96; +2.15)
precordial leads only	V1+V2+V5 (-0.44; +9.54)	V2+V3+V5 (+0.00; +32.34)	V1+V2+V4 (-4.89; +16.22)
must contain lead II	II+V1+V4 (-7.58; +2.40)	II+V2+V5 (-11.08; +21.27)	II+V2+V4 (-2.19; +18.92)

Table S23. Paired patient-clustered contrasts between the reduced-lead models. The three lead-set encoders of Table 6 were retrained with the locked recipe (full-12 teacher, seed 42; macro-AUROC 12 leads 0.901, I+V1+V4 0.878, II+V1+V5 0.887, II 0.844), their per-record C5 probabilities on fold 10 kept together with those of the locked 12-lead model, and the six pairwise macro-AUROC differences estimated by 2000 joint patient-clustered bootstrap resamples with two-sided bootstrap p-values and Benjamini–Hochberg control (protocol of Table S16).

Contrast	AUROC$_a$	AUROC$_b$	Δ	95% CI	p	p_{BH}
12 leads vs I+V1+V4	0.901	0.878	+0.0233	[+0.0140, +0.0324]	< 0.001	< 0.001
12 leads vs II	0.901	0.844	+0.0571	[+0.0432, +0.0718]	< 0.001	< 0.001
II+V1+V5 vs II	0.887	0.844	+0.0428	[+0.0293, +0.0568]	< 0.001	< 0.001
I+V1+V4 vs II	0.878	0.844	+0.0338	[+0.0182, +0.0503]	< 0.001	< 0.001
12 leads vs II+V1+V5	0.901	0.887	+0.0143	[+0.0056, +0.0231]	0.001	0.001
I+V1+V4 vs II+V1+V5	0.878	0.887	-0.0090	[−0.0198, +0.0012]	0.092	0.092

4. Supplementary Notes

Note S1. Record disposition under the clean single-label criterion.

Of the 21,799 PTB-XL records, 946 carry none of the four target superclasses (411 with no diagnostic superclass at all, 535 labeled only with hypertrophy), 3,878 carry more than one target superclass, and 16,975 carry exactly one, of which 1,266 also carry a hypertrophy code and are excluded, leaving 15,709. Retention by class is 95.3% for records carrying NORM, 46.3% for MI, 45.8% for STTC and 34.9% for CD, so the criterion raises the NORM:MI ratio from 1.74 in the full database to 3.58 in the analyzed subset (Section 2.1); its consequences for the MI operating point and case mix are quantified in Sections 3.4 and 4.5 and Table S8.

Note S2. Effective rank of the coupled parameterization.

In the graph (eikonal-inspired) model the activation times are generated by propagation along the conduction graph, so the timing parameters are coupled: proximal edge delays and the root onset act as near-common-mode shifts that are weakly distinguishable from a global time reference. One might expect an unconstrained parameterization with eight free activation times to show a higher effective rank, each time being identifiable from its own nodal shift. The structural ablation of Table S20 measures this in radius-scaled coordinates and finds the opposite: at 12 leads the free-activation-time model has an effective rank of 4.40 against 4.36 for the locked model, and for lead II alone 1.83 against 2.84. Coupling the activation times through the graph is what lets a single lead constrain the whole activation sequence; with free times, a lead that sees only part of the ventricle leaves the other times unresolved, and the ablation also shows that free times violate the activation order of the tree in 98% of test records (Section 3.3). The ordering of lead sets that the main text relies on is that of $\log\det F$ and the Cramér–Rao bounds, which are monotone under lead removal, whereas the effective rank measures spectral spread and is not.
